# Predicting liver cytosol stability of small molecules

**DOI:** 10.1186/s13321-020-00426-7

**Published:** 2020-04-07

**Authors:** Pranav Shah, Vishal B. Siramshetty, Alexey V. Zakharov, Noel T. Southall, Xin Xu, Dac-Trung Nguyen

**Affiliations:** National Center for Advancing Translational Sciences (NCATS), National Institutes of Health (NIH), 9800 Medical Center Drive, Rockville, MD 20850 USA

**Keywords:** Xenobiotic metabolism, Cytosol stability, Matched molecular pairs, Qualitative-structure activity relationship, Machine learning

## Abstract

Over the last few decades, chemists have become skilled at designing compounds that avoid cytochrome P (CYP) 450 mediated metabolism. Typical screening assays are performed in liver microsomal fractions and it is possible to overlook the contribution of cytosolic enzymes until much later in the drug discovery process. Few data exist on cytosolic enzyme-mediated metabolism and no reliable tools are available to chemists to help design away from such liabilities. In this study, we screened 1450 compounds for liver cytosol-mediated metabolic stability and extracted transformation rules that might help medicinal chemists in optimizing compounds with these liabilities. In vitro half-life data were collected by performing *in*-*house* experiments in mouse (CD-1 male) and human (mixed gender) cytosol fractions. Matched molecular pairs analysis was performed in conjunction with qualitative-structure activity relationship modeling to identify chemical structure transformations affecting cytosolic stability. The transformation rules were prospectively validated on the test set. In addition, selected rules were validated on a diverse chemical library and the resulting pairs were experimentally tested to confirm whether the identified transformations could be generalized. The validation results, comprising nearly 250 library compounds and corresponding half-life data, are made publicly available. The datasets were also used to generate in silico classification models, based on different molecular descriptors and machine learning methods, to predict cytosol-mediated liabilities. To the best of our knowledge, this is the first systematic in silico effort to address cytosolic enzyme-mediated liabilities.
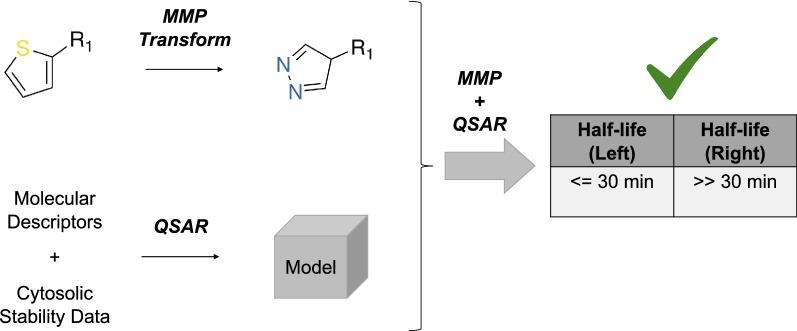

## Introduction

Over the last few decades, chemists have become increasingly proficient at designing compounds that are able to circumvent cytochrome P (CYP) 450 mediated clearance. This has led to the emergence of other drug metabolizing enzymes such as esterases, aldehyde oxidase, etc. as significant contributors to drug clearance [1]. Typical screening assays are performed in liver microsomal fractions as they contain the CYP450 enzymes and it is possible to overlook the contribution of non-microsomal clearance until later stages of the drug discovery process. In 2008, the development of compound SGX523 was discontinued after reports of dose-limiting renal toxicity [2]. Investigational New Drug-enabling studies for SGX523 were performed in dogs and rats since no significant differences were seen in rat, dog, monkey and human microsomal clearance profiles. It was later found that the toxicity occurred due to the accumulation of the aldehyde oxidase (AO)-mediated insoluble toxic metabolite in the kidneys [2, 3]. In the case of compound FK3453, clinical trials had to be terminated due to the abnormally low levels of parent compound in human plasma [4]. The hepatic availability in human was calculated by in vitro-in vivo scaling using in vitro intrinsic clearance data from liver microsomes and in vivo intrinsic clearance data from rats and dogs. AO-mediated metabolism was found responsible for the low exposure of FK3453 in humans [4]. These examples highlight the importance of understanding the metabolism of new drug candidates in systems that contain the full array of metabolizing enzymes.

Several well-known structure modification strategies exist to guide the medicinal chemist through CYP-mediated instabilities [5, 6]. However, strategies to attenuate non-CYP-mediated liabilities are sparsely reported in the literature [7–10]. A few *in*-*house* projects at the National Center for Advancing Translational Sciences (NCATS) were recently discovered to have cytosol-mediated metabolic liabilities. We generated half-life (t_1/2_) data for 1450 compounds using mouse and human cytosol fractions. The aim of this study is to explore these datasets and provide chemists with guidelines which can be used to rationally modulate compounds and attenuate cytosol-mediated metabolic instabilities. To achieve this, we performed matched molecular pairs analysis (MMPA) on the stability data measured in mouse and human cytosol fractions. This concept is widely applied in medicinal chemistry due to the possibility to associate defined structural transformations between compound pairs with corresponding changes in molecular properties [11–13]. Thus, MMPA allows us to extract inherent medicinal chemistry knowledge from these datasets and helps understand the impact of important chemical structure modifications on cytosolic stability [14]. The extracted rules were validated on an *in*-*house* small molecule library and their general tendency to improve cytosolic stability was experimentally validated. A detailed analysis of the structure–metabolism data revealed certain transformations that demonstrated opposite effects on the stability of compounds in human and mouse cytosol fractions. In addition, we present in silico classification models, based on by far the largest dataset reported in the literature, that will help facilitate identification of compounds with potential cytosol-mediated metabolic liabilities.

## Methods and data

### Metabolic stability assay

Mixed gender human (H0610.C) and male CD-1 mouse liver cytosol fractions (M1000.C) were purchased from Sekisui XenoTech, LLC (Kansas City, USA). We chose the substrate depletion method to determine in vitro t_1/2_. Our assay (384-well format) consisted of two parts; a robotic system for incubation and sample clean up and an integrated LC/MS method to calculate the percent remaining of parent compound. Briefly, each reaction mixture (110 μL) consisted of a test compound (1 μM) and either human or mouse cytosol fractions (2 mg/mL) in phosphate buffer (100 mM) at pH 7.4. Samples were incubated in 384-well plates at 37 °C for 0, 5, 10, 15, 30 and 60 min. Sample analysis and t_1/2_ calculations were performed using a previously described method [15]. Using this high-throughput method, we generated t_1/2_ data for 1450 compounds in mouse and human cytosol fractions. A majority of these compounds were part of three on-going *in*-*house* projects. A set of control compounds for low and high cytosolic stability were routinely run in each plate. The reproducibility data for exemplary control compounds used in the assay are presented in Table 1. The minimum significant ratio (MSR) for famciclovir (human and mouse) as well as carbazeran (human) were found to be below 3 which indicates the robustness of the assay.

**Table 1:** Reproducibility across different experiments

Compound	# Repeats^a^	t_1/2_ (mean ± SD)/category (Mouse cytosol)^b^	t_1/2_ (mean ± SD)/category (Human cytosol)^b^
Buspirone	17	>120 min/High	>120 min/High
Antipyrine	17	>120 min/High	>120 min/High
Famciclovir	17	9.5 ± 3.2 min/Low	10.0 ± 3.1 min/Low
Carbazeran	17	>120 min/High	3.5 ± 1.8 min/Low

^a^t_1/2_ values of the control compounds across 17 plates were measured by UHPLC/HRMS. t_1/2_ categories: < 10 min: low; > 30 min: high

^b^No SD values provided if all replicate t_1/2_ values exceeded 120 min

### Training data

The set of 1450 compounds tested in cytosolic stability assay (human and mouse) is considered as the training set. For the purpose of this study, we distinguish unstable compounds from stable compounds using the following criteria: t_1/2_ ≤ 30 min—unstable; t_1/2_ > 30 min—stable. The chemical structures of the compounds were standardized, and the normalized structures were used to generate standard InChIKey notations. The steps involved in standardization of chemical structures are detailed in Additional file 1: Table S1. Entries sharing the same standard InChIKey were marked as duplicates and the compound was retained only if all corresponding entries belonged to the same class (i.e., stable or unstable). Finally, the training sets from both human and mouse cytosol fractions consisted of 1230 unique compounds.

### Matched molecular pairs analysis setup

A matched molecular pair can be defined as a pair of compounds that differ only at a single site through a well-defined structural transformation that is associated with a relative change in a property value [11, 13, 16]. In this study, MMPA was performed using the open-source KNIME Analytics Platform (version 3.7) [17] that facilitates automatization of data integration and analysis for a range of cheminformatics tasks. The Vernalis KNIME nodes [18], that reproduce the Hussain and Rea algorithm [16], were used to perform MMPA in addition to different RDKit [19] community nodes. The steps involved are molecule fragmentation, identification of matched pairs and compilation of unique transformation rules. In order to prospectively validate the transformations, training dataset was further divided into two parts, i.e., training and test set, at a ratio of 70:30 in a random manner. The same random seed was used for both human and mouse data so that their training and test set compositions are similar. The KNIME workflow for MMPA can be found in the supporting material (Additional file 2).

### Cytosolic stability prediction model

Next, we sought to develop in silico classifiers based on the human cytosol stability data to facilitate identification of potential substrates during the lead optimization phase. Since the mouse data was found to be heavily skewed towards the stable class, we decided to build a predictive model based on the human dataset. The same dataset used for the generation of MMP rules was employed for this purpose. Binary classifiers were built using Random Forests (RF) [20], Support Vector Machines (SVM) [21, 22] and BayesNet [23] approaches. RF and SVM algorithms were chosen on the basis of their popularity in predicting molecular properties [24]. BayesNet node, made available in KNIME by WEKA, facilitates learning using different Bayes Network learning algorithms. The method was previously employed by Zakharov et al. [25] in predicting metabolic stability of compounds in human liver microsomes and was reported to be among those methods that provided higher sensitivity in predicting unstable compounds. Three sets of molecular descriptors were independently used as features: two-dimensional (2D) descriptors available from the Molecular Operating Environment (MOE; Chemical Computing Group ULC.) [26]; 2D descriptors calculated from the RDKit Descriptor Calculation node in KNIME; and QNA descriptors [27, 28]. The descriptors from MOE and RDKit were normalized, a pair-wise correlation analysis was performed, and the most-correlated (r^2^ > 0.9) and constant value descriptors were removed, which left 119 MOE descriptors (out of 206) and 80 RDKit descriptors (out of 119). A total of 120 QNA descriptors include 10 whole molecule 2D descriptors and 110 QNA descriptors accounting for ionization potential and electronic affinity of individual atoms based on a connectivity matrix representation [28]. The QNA descriptors were not subjected to correlation analysis and normalization due to their orthogonal nature but any constant-value descriptors were omitted. These descriptors were selected as they were previously reported to perform well in the prediction of t_1/2_ values in human liver microsomes [25]. In addition to the models based on RF, SVM and BN, we built a deep learning model based on a directed message passing neural network recently proposed by Yang et al. [29] for molecular property prediction. It is a graph convolutional neural network (GCNN) that operates on graphs structures of molecules to construct a learned molecular representation. This model was selected on the basis of its competitive performance on a large number of datasets as compared to the publicly available benchmark from Wu et al. [30].

**Fig. 1:**
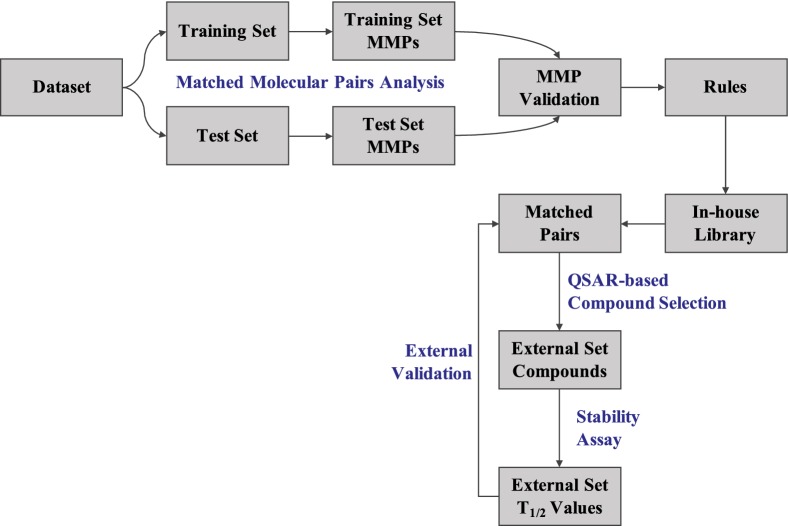
A schematic representation of the study methodology

Since the training set was skewed towards the majority class (i.e., stable compounds), we generated balanced training sets by under-sampling the majority class. Two different methods were employed: diversity under-sampling and multiple under-sampling. In the first method, chemically diverse compounds were picked from the majority class using the RDKit diversity picker node which uses molecular fingerprints to pick an arbitrary number of diverse compounds. The number of compounds picked is equal to the number of compounds in the minority class (i.e., unstable compounds). In multiple under-sampling, majority class instances are randomly sampled to the size of minority class and this was repeated until each majority class instance has become part of the training set at least once. Average of the individual models that are based on distinct subsets of majority class were reported as the final performance values [31]. The models were evaluated using different performance metrics. An overview of these metrics can be found in Additional file 1: Table S5. A complete workflow that represents the methodology is shown in Fig. 1.

### MMP and QSAR model: external validation

The transforms from the training sets were applied to the test sets in a prospective manner. Further, selected transformations from the training set were validated on an independent external dataset (*in-house* library consisting of 96,358 compounds) using the ‘RDKit Apply Transformations’ node. The library comprises a large number of core scaffolds, representing 20 to 100 compounds per chemotype that facilitate optimization efforts in medicinal chemistry projects and therefore fits well with the scope of the study. The node takes a list of transformations and a set of molecules as input and provides an additional column containing the transformed molecules. We then checked if the transformed molecules were present in our library to generate a list of matched pairs that obey the rules derived from the training set. These independent compound pairs were tested in the same cytosolic stability assay to assess if the transforms improve cytosolic stability.

For human dataset, we obtained a large number of such pairs. In order to select a limited number of pairs for experimental validation, we picked those matched pairs in which the compound on left was predicted as unstable by the QSAR model based on training set. A total 160 compounds (80 matched pairs) were chosen for experimental validation. Once the stability data was generated (we could obtain data for only 155 compounds), we used the same set for validating the QSAR model. Therefore, this set of 155 compounds is considered as external validation set. For the mouse dataset, only 29 pairs (58 compounds) were found in the library and we tested all 29 pairs as part of the external validation.

## Results

### Molecular properties and data distribution

**Fig. 2:**
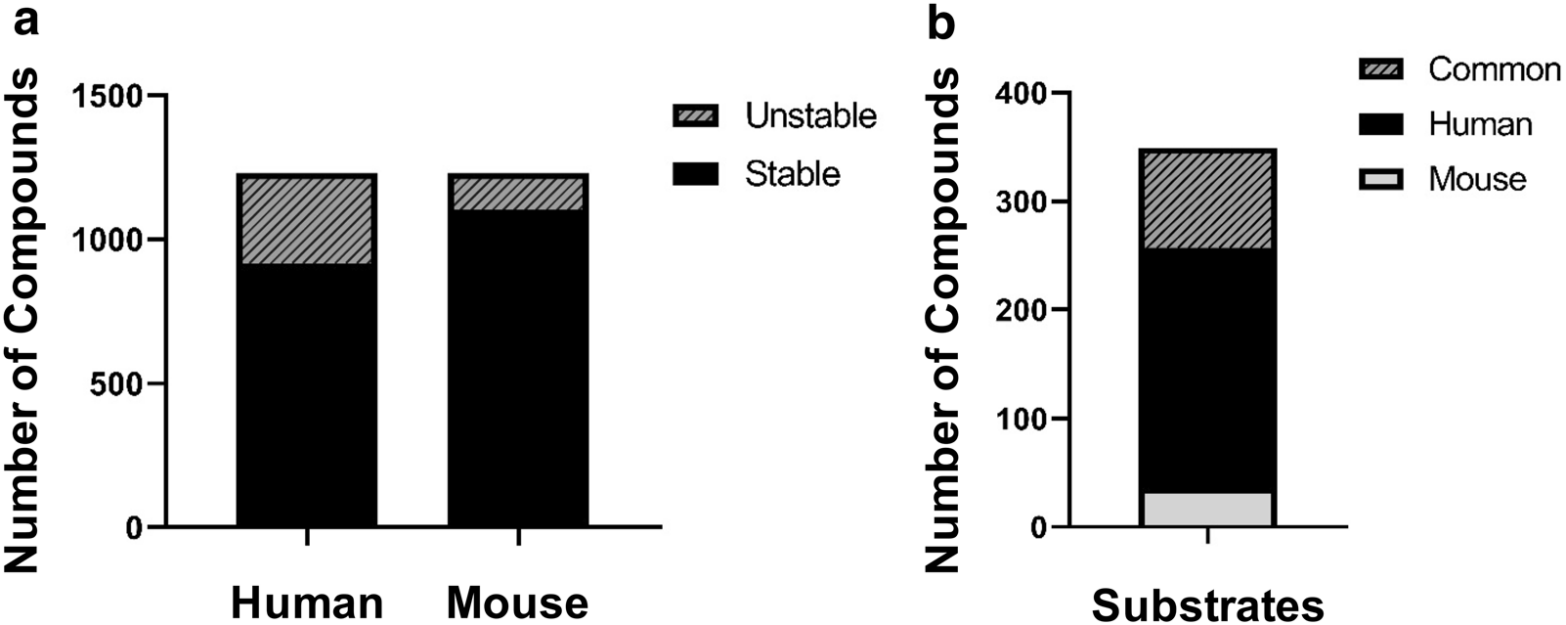
**a** Distribution of training set compounds from human and mouse datasets. **b** Overlap of substrates across datasets

**Fig. 3:**
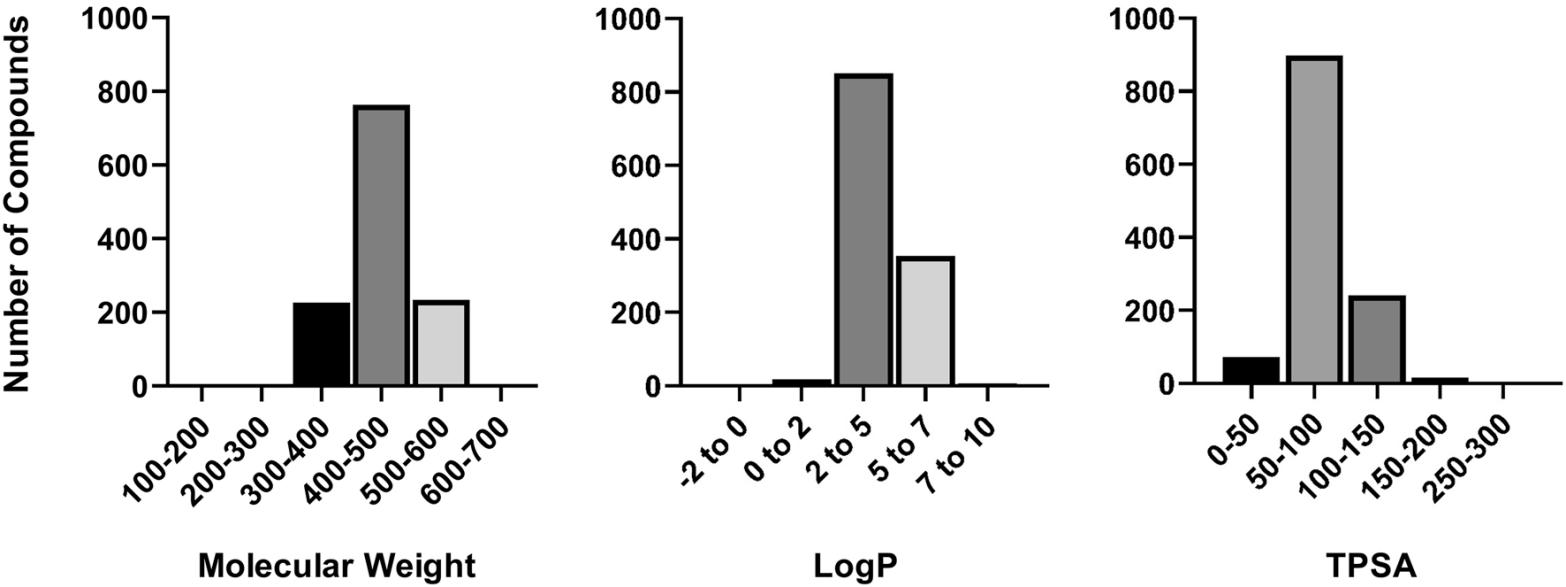
Distribution of training set compounds on the basis of molecular properties

From the distribution of simple molecular properties such as log P, topological polar surface area (TPSA) and molecular weight (MW) for the training set compounds (Figs. 2, 3), a majority of compounds have t_1/2_ values greater than 30 min in human and mouse cytosol fractions, belong in the 300–500 MW range, have TPSA < 100 and log P values ranging from 2.0 to 5.0. No direct correlation could be detected between the experimental t_1/2_ values and the calculated molecular properties. Furthermore, significant differences in the distribution of t_1/2_ values were observed between the mouse and human datasets. Of the 1230 compounds, 313 (25.4%) compounds were classified as unstable (t_1/2_ < 30 min) in human dataset as compared to 128 (10.4%) in the mouse dataset. 92 compounds were found to be unstable in both mouse and human datasets (Fig. 2 a, b).

### MMPs from training data (prospective validation)

**Fig. 4:**
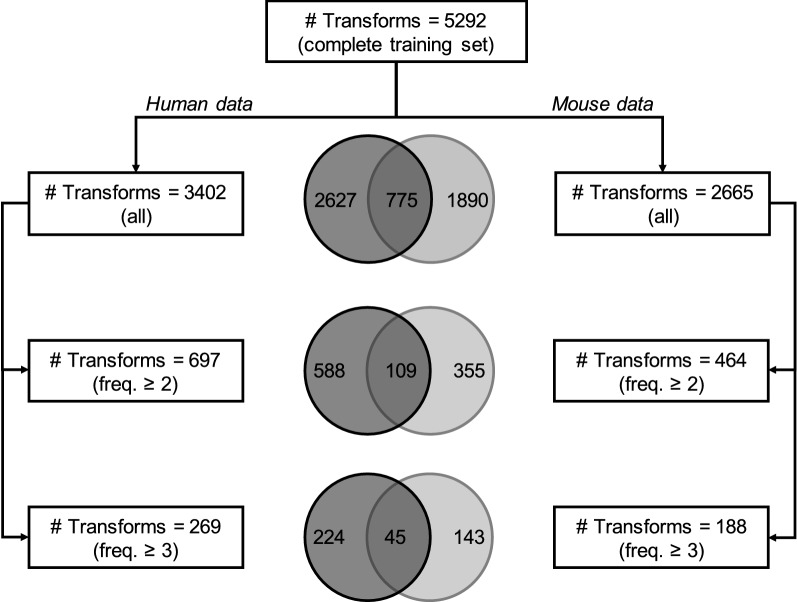
Statistics on the MMPs obtained using the prospective training sets

**Fig. 5:**
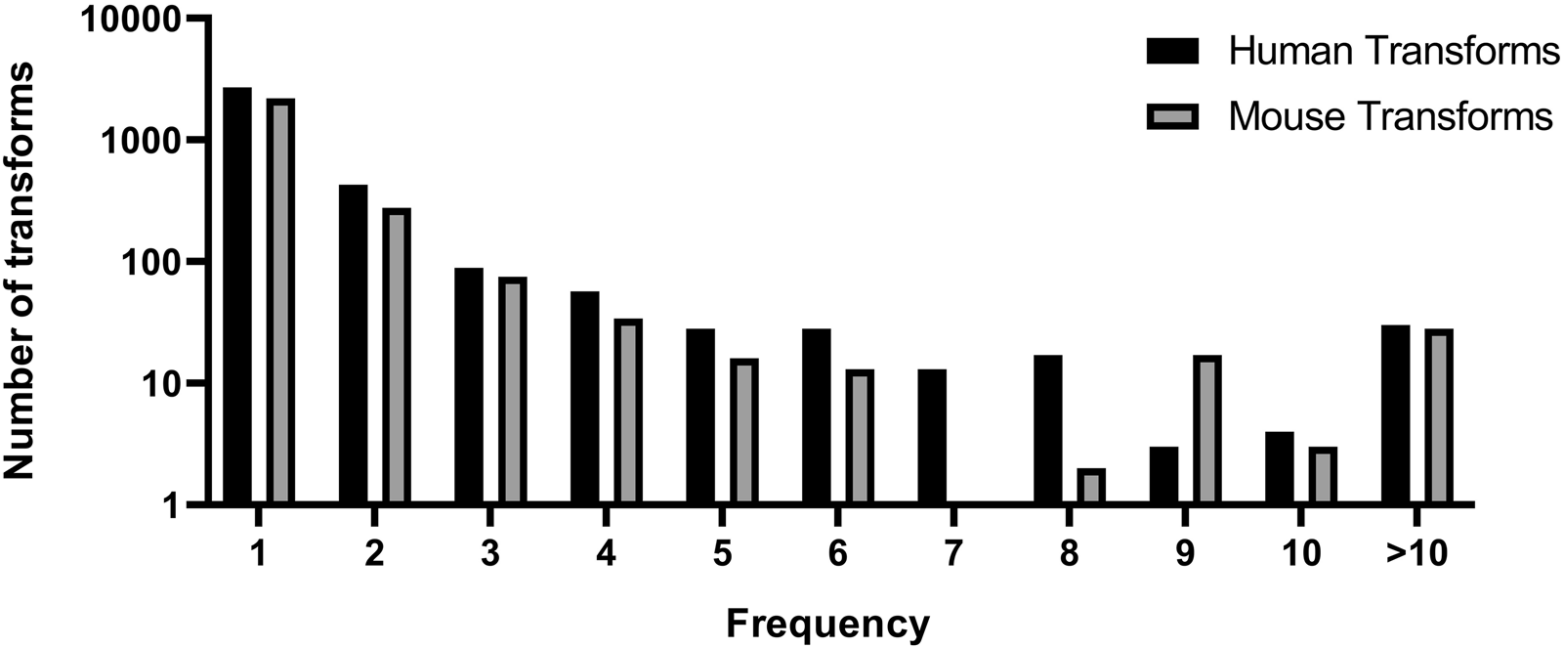
Distribution of transforms from human and mouse datasets based on frequency

Compounds from the training set were processed in the MMP workflow. Prospective training set and test set from both species were treated exactly the same. Unique transformations were generated from the training and test sets, followed by identification of a list of transformations common to both these sets. This serves as prospective validation of the list of common transforms as though they were selected from the training set and applied on the test set compounds for validation. The training sets comprised of 1100 (human dataset) and 1105 (mouse dataset) compounds which represent 70% of the corresponding individual training sets. The remaining 30% portion served as a prospective test set. A total of 5292 unique transforms were identified from the two training sets. The overlap of training set transforms from human and mouse data, and their frequencies are presented in Figs. 4 and 5. It is clear that the human training set provided more transformations. However, the difference was less pronounced when only transforms with at least two or three examples each were considered. Similarly, the number of transforms in common decreased with the frequency criteria. Overall, the transforms from human dataset were more frequently found as compared to the transforms from mouse dataset. Since the compounds demonstrated different behaviors in mouse and human cytosol fractions, the number of transforms from both these datasets differ.

As part of the validation, 94 human transforms were applied on 46 test set compounds. In 50% of the cases, the t_1/2_ increased by at least 1.5-fold. Only 12 out of the 46 compounds were unstable. The transformations could successfully transform nine compounds from unstable to stable. Similarly, 44 transforms from mouse dataset were applied on 20 prospective test set compounds. In at least 50% of the cases, the t_1/2_ could be increased by at least 1.5 times and five out of the seven unstable compounds were successfully transformed into stable analogues. The top 10 transformations form both datasets that could successfully transform unstable compounds to stable are listed in Additional file 1: Tables S2, S3.

### Species differences

It is interesting to note that the mouse dataset contains more stable compounds than the human dataset. As pointed out earlier, clinical development of some drug candidates were terminated due to their unfavorable metabolic profiles in human that were not predicted during preclinical studies [32, 33]. This could explain why the dataset is more skewed toward the ‘stable’ class in the mouse dataset. Further investigation led to the identification of some transforms that showed opposite effects in human and mouse (see Table 2). Here, we consider only those examples in which the compound pairs involved in the transformation are identical, i.e., the same compound pair demonstrates a positive shift in mouse and a negative shift in human or vice versa. This analysis was beyond the scope of the study and we plan to pursue this further as part of a future study.

**Table 2:**
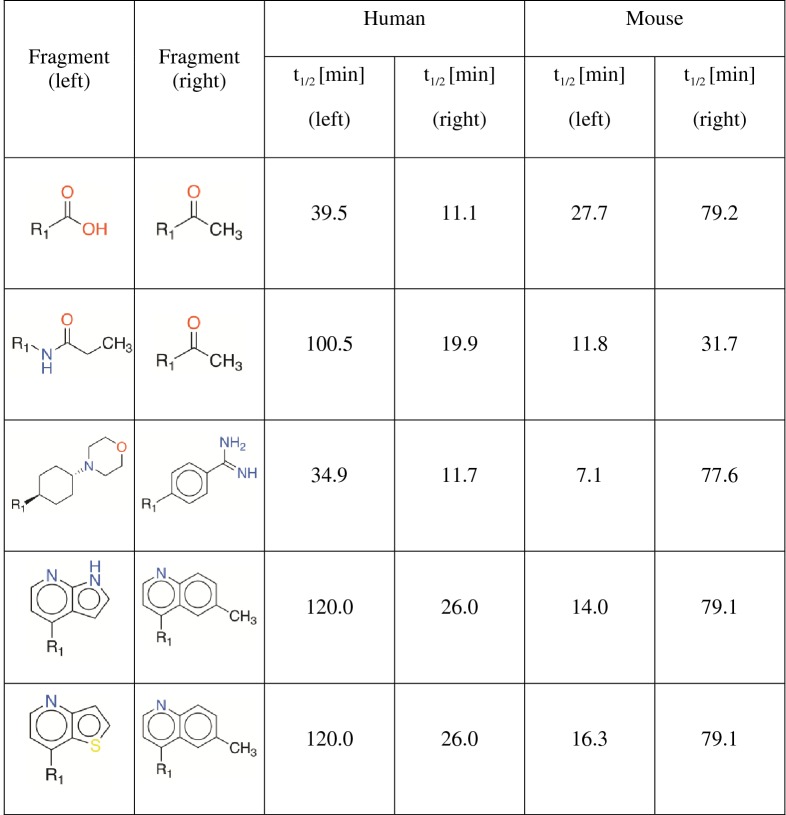
MMP transforms that demonstrated opposite behavior in human and mouse cytosol fractions

### Cytosolic stability prediction models

The primary reason behind building a QSAR model based on this dataset was to prioritize the compound pairs that resulted from application of MMP rules identified in the first part of the analysis. However, we pursued it further to explore if the dataset in hand could be of any use to develop computational models to identify potentially unstable compounds in cytosolic fractions. During cross-validation (see Table 3), SVM-based classifiers performed on average better as compared to the classifiers based on RF and BN approaches. Although the AUC and BACC values did not differ significantly between SVM and RF models, SVM tended to provide higher Sensitivity (i.e., the ability to correctly predict unstable compounds) while RF provided higher Specificity. Although the BN models provided inferior BACC values in general, they provided a balance between Sensitivity and Specificity. No significant differences in performance could be detected in terms of the different descriptors. As described earlier, we assessed the influence of under-sampling the majority class on model performance. In case of RF classifiers, the training set based on multiple under-sampling clearly provided better BACC with Sensitivity being the major contributor. While no consistent trend could be detected for BN classifiers, the SVM classifiers demonstrated an improvement with both down-sampling approaches. RF and SVM models banked more from under-sampling the majority class. The best performing model was an SVM classifier based on diversity under-sampling method and QNA descriptors with BACC, Sensitivity and Specificity of 0.8 each and an AUC of 0.85.

**Table 3:** Cross-validation results

Descriptor	Method^a^	AUC-ROC	BACC	Sensitivity	Specificity
MOE	BayesNet—DU	0.81	0.76	0.72	0.79
	RF—MU	0.86	0.78	0.79	0.76
	SVM—DU	0.85	0.77	0.79	0.76
RDKit	BayesNet	0.79	0.73	0.61	0.85
	RF—MU	0.86	0.78	0.78	0.78
	SVM—DU	0.83	0.77	0.83	0.72
QNA	BayesNet—MU	0.75	0.69	0.62	0.75
	RF—MU	0.83	0.75	0.72	0.78
	SVM—DU	0.85	0.80	0.80	0.80

^a^DU and MU stand for diversity under-sampling and multiple under-sampling, respectively

### QSAR validation

In external validation, surprisingly, the SVM classifiers demonstrated a poor performance. BayesNet classifiers always provided the best performance based on the original training set. In majority cases, diversity under-sampling resulted in a poor performance due to a drop in Sensitivity. RF classifiers based on MOE descriptors were least affected by this. Although BN classifiers provided higher Sensitivity values, the highest BACC was achieved by the RF classifier based on MOE descriptors and original training set. Again, no significant differences in performance could be found between the models on the basis of different descriptors. However, in case of SVM and BN, QNA descriptors provided a better balance between Sensitivity and Specificity. When using MOE or RDKit descriptors, these models tended to predict all compounds as stable. In Tables 3 and 4, for each descriptor and method, only the performance of the best model (out of the three models: no sampling, diversity under-sampling, multiple under-sampling) was provided. The complete results for cross-validation and external validation are provided in Additional file 1: Table S6 and Table S7. In the light of recent advancements in development of novel molecular representations for deep learning, molecular graphs have been claimed to outperform traditional descriptors in predicting chemical properties and biological activities [29, 30]. These studies employed graph features as descriptors to perform QSAR modeling on large number of datasets from different domains using the GCNN architecture. Therefore, we employed the most recent version [29] of this algorithm for prediction of cytosol stability of the validation set compounds. Surprisingly, the GCNN models did not perform better than any classical machine learning algorithm employed in this study.

**Table 4:** External validation results

Descriptor	Method	AUC-ROC	BACC	Sensitivity	Specificity
MOE	BayesNet	0.62	0.60	0.75	0.45
	RF	0.73	0.64	0.63	0.66
	SVM—MU	0.51	0.56	0.35	0.86
RDKit	BayesNet	0.56	0.57	0.50	0.64
	RF—MU	0.68	0.61	0.71	0.52
	SVM—MU	0.62	0.54	0.15	0.94
QNA	BayesNet	0.63	0.62	0.63	0.60
	RF—DU	0.50	0.58	0.25	0.89
	SVM—MU	0.60	0.57	0.63	0.52
Graphs	GCNN	0.48	0.53	0.38	0.68

We then investigated the reason behind the inferior performance of the models in external validation. Principal component analysis (see PCA plots in Additional file 1: Figure S1) revealed that the chemical space of the training set is homogeneous, and the compounds are distinct from the external dataset which was derived from a large library of compounds representing diverse chemotypes. This suggests that most external set compounds do not fall within the applicability domain of the training set used to develop the models, limiting their applicability to a new dataset. We believe that a consensus of the best-performing individual models might improve the prediction performance on the external dataset. Since the goal was not to identify the best QSAR model, we did not investigate further into improving the models reported in this study. The modeling methods and corresponding parameters are listed in Additional file 1: Table S4.

### MMP validation

A total of 10 transforms (7 transforms from human data; 3 transforms from mouse data) were applied on *in*-*house* library of compounds. We prioritized testing compound pairs that had the largest difference in predicted stability to identify pairs where the compound on the left is potentially unstable. This would allow evaluation of the ability of the chemical transforms to improve cytosolic stability. A quantitative structure–activity relationship (QSAR) model (*see previous section for more details*) was employed to predict the cytosolic stability of all compounds on left and 96 compound pairs were prioritized for testing in the metabolic assay. In summary, a total of 7 human transforms (96 compound pairs) and 3 mouse transforms (29 compound pairs) were experimentally validated by determining the t_1/2_ of the compounds in the very same cytosol stability assay. The number of compound pairs per transformation and their effects on the *in*-*house* library compounds are summarized in Table 5 and representative results are shown in Table 6. The complete validation results containing chemical structures of the 125 (96 + 29) compound pairs and their t_1/2_ values from the stability assay are provided in the supporting material (Additional file 3). Due to the proprietary nature of the dataset, we could not provide the structures of the training set. However, exemplary transformations are provided in Additional file 1: Tables S2 and S3.

**Table 5:**
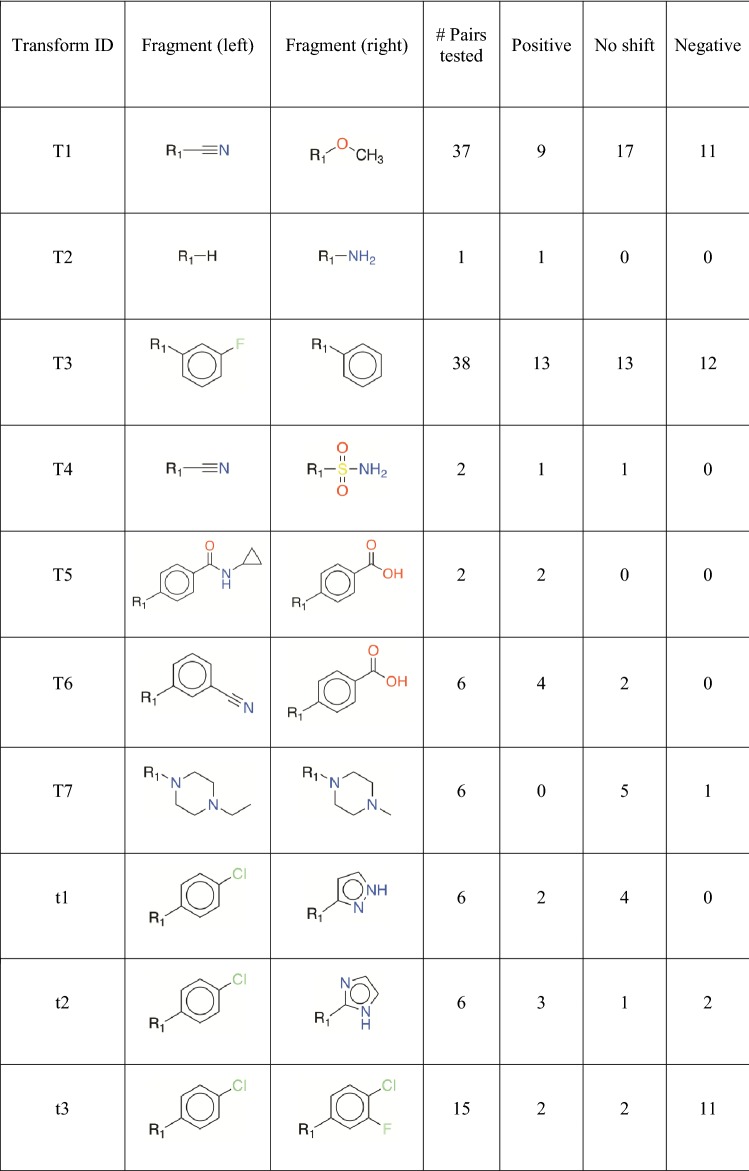
A summary of the effects of the MMPs selected for external validation^a^

^a^T—transforms are based on human data; t—transforms are based on mouse data

**Table 6:**
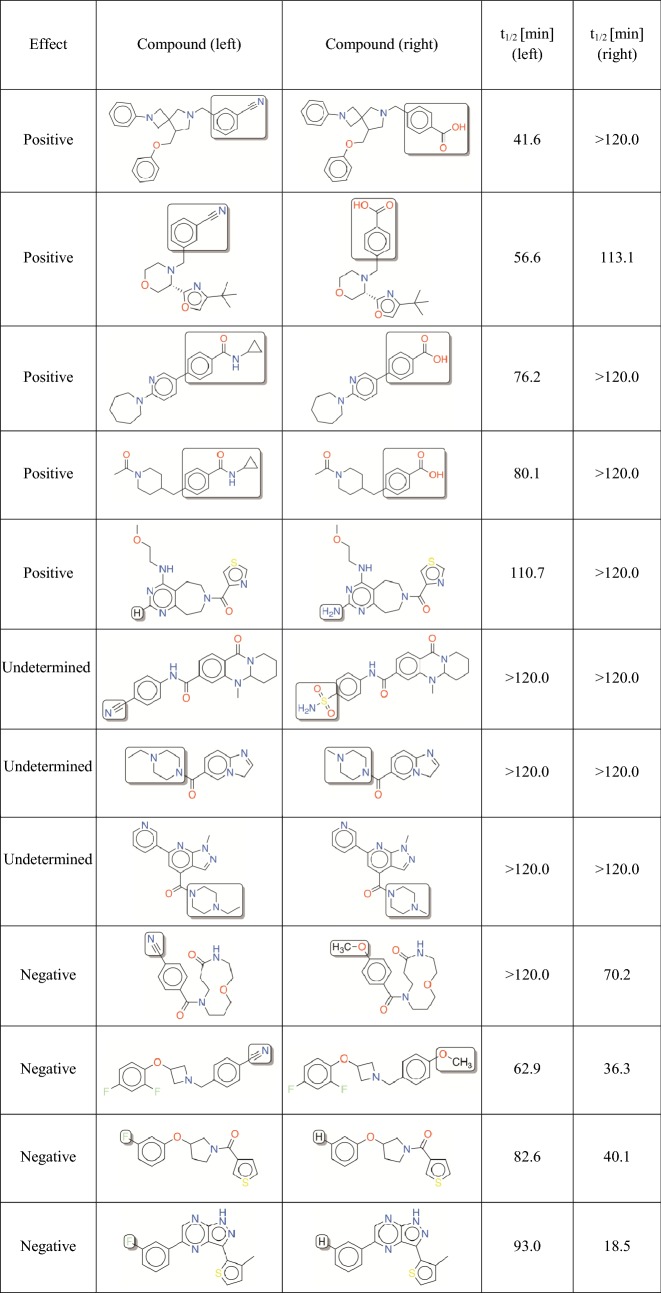
Compound pairs from the *in-house* library that exemplify the effects of experimentally validated MMP transforms

While the human transforms were represented by distinct fragments on both left and right sides, all mouse transforms had the same fragment on the left side. In either case, the number of compound pairs per transform varied, with some representing more than 30 pairs and some representing only one or two pairs of compounds. For instance, the amine substitution represents only one out of the 96 pairs tested although the set of 1166 compound pairs comprises another 45 pairs represented by this transform. The very same transform represents 53 compound pairs in the training set with an average shift of 62.3 (± 51.4) min, which indicates that on average the t_1/2_ increased by 62 min. The geometric mean of the shift in t_1/2_ was found to be 32 min. Due to the lack of adequate number of validation examples per transform, we did not decide upon explicit criteria to identify significant transforms. Overall, the transforms demonstrated mixed effects. Few transforms resulted in ‘positive shift’ and ‘no shift’ while others demonstrated a ‘negative shift’.

Out of the 96 compound pairs based on human transforms, two pairs had to be excluded from the analysis due to lack of mass-spectrometry signal. We identified seven pairs from the remaining 94, for which the compound on left-hand side was unstable. Three of these compounds (t_1/2_ = 8.2, 17.4 and 17.4 min) could be successfully transformed into stable compounds (t_1/2_ = 35.6, 93.0 and 38.3 min). We observed no change in t_1/2_ values for remaining four unstable compounds. However, a total of 17 pairs demonstrated 1.5-fold increase in t_1/2_. While, a total of nine pairs from total 96 demonstrated ‘significant negative shift’, we could not conclude the effect of transforms on 40 pairs since the compounds on both left- and right-hand side demonstrated a t_1/2_ value of > 120 min. Since the t_1/2_ values could not be extrapolated beyond 120 min, we consider the effect of transforms on these pairs as inconclusive or undetermined.

Only two out of 29 compound pairs based on mouse transforms comprised of an unstable compound on the left-hand side. Of the two, one pair showed a ‘positive shift’ and the other one showed ‘no shift’. Two pairs out of 29 total pairs demonstrated a ‘significant negative shift’, while seven were inconclusive or undetermined. The t_1/2_ improved by more than 1.5-fold only in case of four pairs. In both human and mouse datasets, few compound pairs demonstrated a ‘shift’ that could not be categorized as either positive or negative, i.e., the t_1/2_ values were within the standard error of the assay. The poor performance of mouse transforms could be explained by the greater skewness of the training set towards stable class in comparison to the human dataset. Example compound pairs from the *in*-*house* library demonstrating positive or negative effects (based on human transforms) are listed in Table 6. The whole dataset is listed as part of supplemental.

## Discussion

Optimization of different pharmacokinetic and toxicity endpoints is fundamental to efficient drug discovery [34]. CYP-mediated metabolism represents a major route of elimination of many drugs [35–38]. Significant progress has been made in reducing metabolic instabilities due to CYP-mediated oxidation [39]. A majority of these low microsomal turnover compounds could be potential substrates for non-CYP metabolism [38, 40, 41]. Many phase I drug metabolizing enzymes are present in the cytosol including aldehyde oxidase, alcohol dehydrogenase, xanthine oxidase (XO), esterases, amidases/peptidases and aldo–keto reductases [42]. Since we did not fortify our assay with additional co-factors, majority of our high clearance compounds would be substrates of either AO, XO, amidases/peptidases or esterases. ~ 7% of our total dataset and 4% of substrates (t_1/2_ < 60 min) contain ester or amide groups (see Table 7). This points toward a major role of AO- and XO-mediated metabolism in our datasets. AO and XO belong to a family of molybdenum-containing enzymes and these enzymes exhibit an extraordinary degree of amino acid sequence similarity. Since the process of making human cytosol fractions involves the use of allopurinol (a potent XO inhibitor), human cytosol fractions are typically devoid of any XO activity [43]. Thus, it is possible that the majority of human transform rules are derived from substrates of AO. In case of the mouse dataset, it is likely that the transform rules are derived from AO and XO substrates.

**Table 7:** Esters and amides among the substrates ( t_1/2_< 60 min) in the training set

Dataset	# Substrates	Amide matches (% substrates)	Ester matches (% substrates)
Human	505	14 (2.8%)	11 (2.2%)
Mouse	235	8 (3.4%)	4 (1.7%)

Matched molecular pairs analysis has been systematically applied in both academia and industry to extract knowledge from small molecule databases with measured properties and bioactivities [14, 34, 44]. We used this computational approach to identify chemical transformations with a potential to affect cytosolic stability. The human dataset provided more transformations as compared to the mouse data. This was contradictory to our expectations since mice have four AO-isozymes compared to only one in humans. We identified that none of the transformations based on mouse data improved cytosolic stability in general, partly due to the skewed nature of the training set (ratio of unstable to stable ~ 0.1). Although some transformations based on human data resulted in a positive shift, these are small numbers when compared to the number of examples available for molecular transformations in the industry catalogs which are derived from large compound collections [34, 45, 46]. Since this was anticipated, our criteria for identifying significant transformations were not too strict. As long as all example pairs show a ‘positive shift’ from left to right, the transformation was considered valuable. We made an exception in case of those transformations that demonstrated ‘no shift’ and considered them useful only when they demonstrated a positive shift and no ‘negative shift’. Those transformations representing at least as many ‘negative shift’ examples as the ‘positive shift’ examples were not considered useful. In case there were more ‘no shift’ examples compared to the ‘negative shift’ examples, the transformations were considered neutral. For all transforms resulting in either ‘positive shift’ or ‘no shift’, we identified the compound pairs in the training set and examined if the pattern remains the same. The mean t_1/2_ values of the compounds from the training set involved in these transformations are listed in Table 8. Although they always demonstrated a positive shift, the limited number of examples impede the chances of obtaining consistent trends. Transformations with at least two compound pairs represent nearly 20% of the total unique transforms, while those representing 10 or more pairs were only 1%. Therefore, we plan on testing additional compounds as a follow-up to validate the general applicability of these transformations.

**Table 8:** Stability data for the training set compounds involved in ‘positive’ and ‘no shift’ transformations

Transform ID	# Training examples	Avg. t_1/2_ [min] (left)	Avg. t_1/2_ [min] (right)	Avg. of Δ t_1/2_ [min]
T2	53	20.7 ± 33.4	83.0 ± 47.0	32.0
T4	3	20.8 ± 9.8	116.2 ± 6.6	94.9
T5	1	19.2 ± 0.0	> 120.0	N/A
T6	3	24.4 ± 5.1	> 120.0	95.5
T7	2	4.0 ± 0.0	96.7 ± 33.0	89.7

The geometric mean of Δ t_1/2_ is provided in addition to the average of t_1/2_ of compounds on left and right

Several computational models of metabolic stability are available in the literature. Most of them focused on predicting metabolic stability in human liver microsomes, indicating that these models are predictive of CYP450-mediated metabolism [25, 47–50]. While some experimental and in silico efforts attempted to predict non-CYP450 mediated clearance (e.g., aldehyde oxidase metabolism) of drugs, most dealt with only a handful of compounds [51–55]. Few studies relied on descriptors such as energy of the tetrahedral intermediate and steric hindrance to develop in silico prediction models [52]. Such descriptors cannot be computed in silico for a large number of chemical compounds. Therefore, we focused on generating QSAR models using molecular descriptors that are routinely used in cheminformatics modeling exercises. Furthermore, recent studies [56, 57] reported that QSAR efforts can be augmented with MMPA. In this modeling strategy, the effects of transformations generated using MMPA are subsequently evaluated using QSAR predictions. In the first study, Warner et al. reported the use of aggregation effect of multiple transformations to generate novel compounds with improved predictions as compared to models based on nearest neighbors [58] and random forests [56, 59]. Similarly, Springer et al. [57] reported delta-models trained on MMPs to predict activity change resulting from a particular chemical transformation. More recently, Keefer et al. [46] applied an opposite strategy to evaluate the effect of chemical transformations on lipophilicity and metabolic clearance. Here, they employed QSAR techniques first to predict activities for compounds with no experimental data. Later, MMPA was performed to evaluate the effect of chemical transformations on the basis of predicted activities. We employed the same strategy in a different way. We applied the selected transformations on a large chemical library with diverse compounds and then used a QSAR model to identify pairs that could be tested. To the best of our knowledge, the QSAR models reported in this study are the first in silico models that predict cytosolic stability. We could not identify molecular properties (i.e., MW, TPSA, log P) that correlate with this pharmacokinetic endpoint of emerging importance, but we reported models based on different sets of molecular descriptors. Further assessment on the validation set compounds revealed that only a fraction of them fall within the applicability domain of the training set compounds. Again, lack of a dataset with structurally diverse compounds hinders development of a robust classifier that should identify compounds with potential cytosol-mediated metabolic liabilities. Therefore, efforts are needed to generate stability data for a diverse collection of compounds in order to generate generally applicable transforms and improve the models reported in this study.

## Conclusion

Little knowledge exists on how to avoid cytosolic enzyme-mediated metabolism of small molecules in comparison to xenobiotic metabolism by microsomal enzymes such as CYP450s. In this study, we generated structure–metabolic stability data for 1450 compounds in order to derive chemical transformation rules that could help medicinal chemists overcome cytosolic metabolism liabilities. Matched molecular pairs analysis was performed to identify rules that were validated on an independent chemical library. Further experimental validation was performed to assess the validity of these rules. To the best of our knowledge, this study reports the first QSAR models for prediction of cytosolic stability of small molecules. Additionally, we release t_1/2_ data for about 250 diverse compounds from an *in-house* library to encourage the community to further develop and validate models for this increasingly important pharmacokinetic endpoint.

## Supplementary information

**Additional file 1.** Additional figures and tables.

**Additional file 2.** MMPA KNIME workflow.

** Additional file 3.** External validation results.

## Data Availability

Compounds used in the training dataset come from active projects at NCATS and the data generated from those compounds cannot be made public currently. However, the dataset supporting the conclusions of this article is included in the additional material in a machine-readable format.

## Abbreviations

2D: Two-dimensional
AO: Aldehyde oxidase
AUC-ROC: Area under the ROC curve
BACC: Balanced accuracy
CYP: Cytochrome P450
FN: False negatives
FP: False positives
MMPA: Matched Molecular Pairs Analysis
MOE: Molecular Operating Environment
MW: Molecular weight
QSAR: Quantitative-structure activity relationship
TPSA: Topological polar surface area
TN: True negatives
TP: True positives
XO: Xanthine oxidase
