# Supplementary material for: Predicting liver cytosol stability of small molecules
Source: J Cheminform. 2020 Apr 7;12:21. doi: 10.1186/s13321-020-00426-7 (PMC7140498; doi:10.1186/s13321-020-00426-7)
Supplement: Supplementary file 1 — Additional file 1. Additional figures and tables. [file 13321_2020_426_MOESM1_ESM.docx]

**Additional Information**

**Table S1**. Steps involved in the standardization of chemical structures before performing MMPA and QSAR modeling. The following steps were performed using ChemAxon’s InstantJChem Version 19.8.0.

| **Step** | **Objective** |
| --- | --- |
| 1 | Removal of salts and water molecules |
| 2 | Aromatization of molecules |
| 3 | Transformation of adjacent positive and negative charges into double/triple bonds |
| 4 | Addition of explicit hydrogens |

**Table S2**. Top 10 chemical transformations from human training dataset that successfully transformed an unstable compound in the test dataset to a stable compound.

| **Left Fragment** | **Right Fragment** | **t_1/2_ [min] (Left)** | **t_1/2_ [min] (Right)** |
| --- | --- | --- | --- |
| 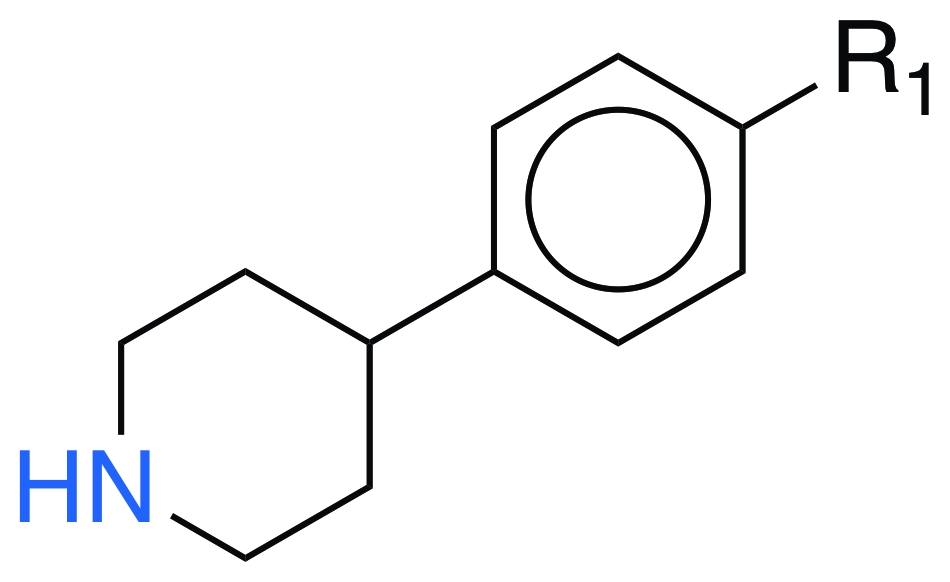 | 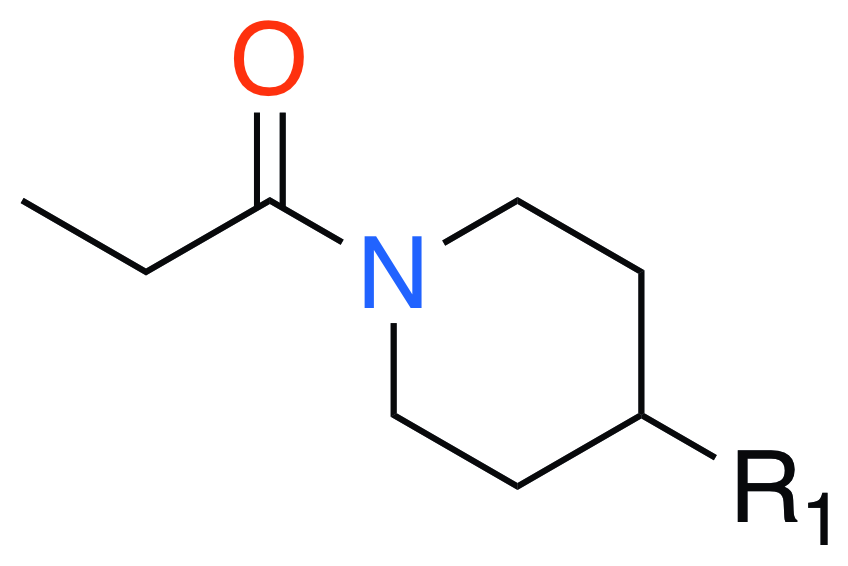 | 3.2 | >120.0 |
| 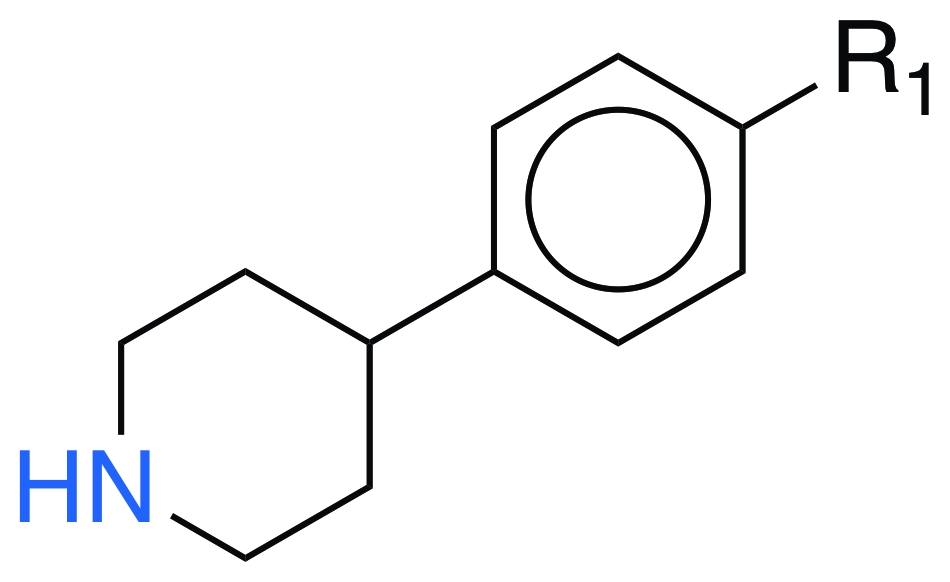 | 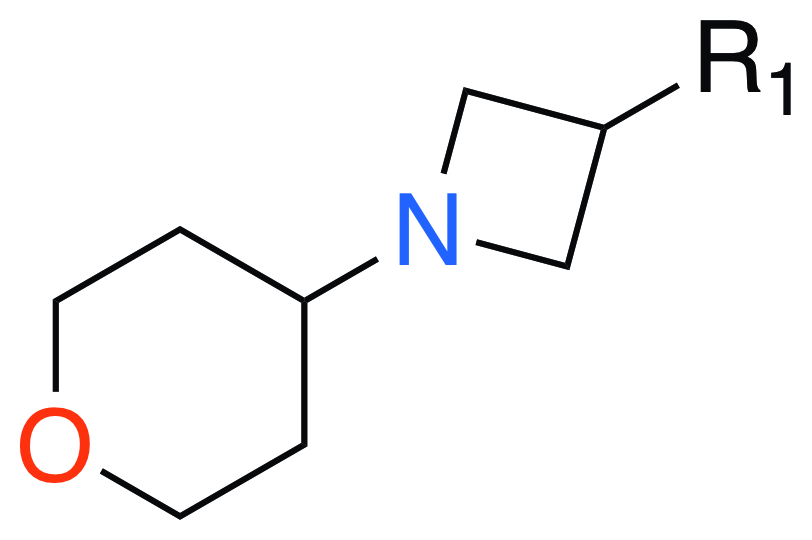 | 3.2 | >120.0 |
| 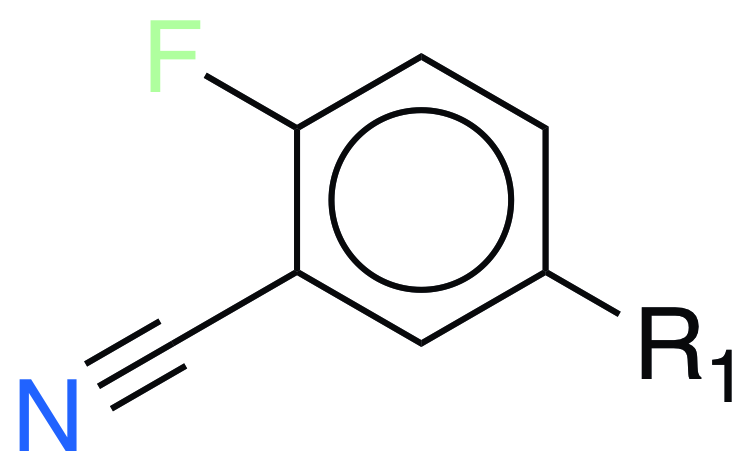 | 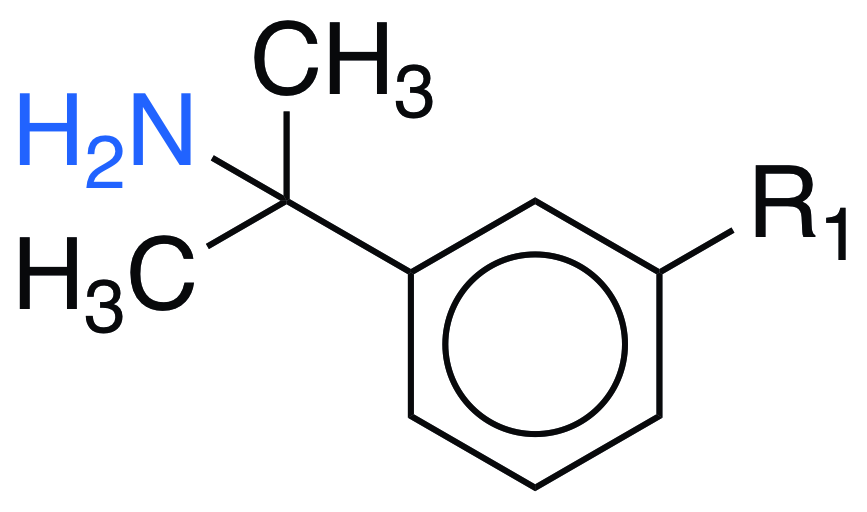 | 3.8 | >120.0 |
| 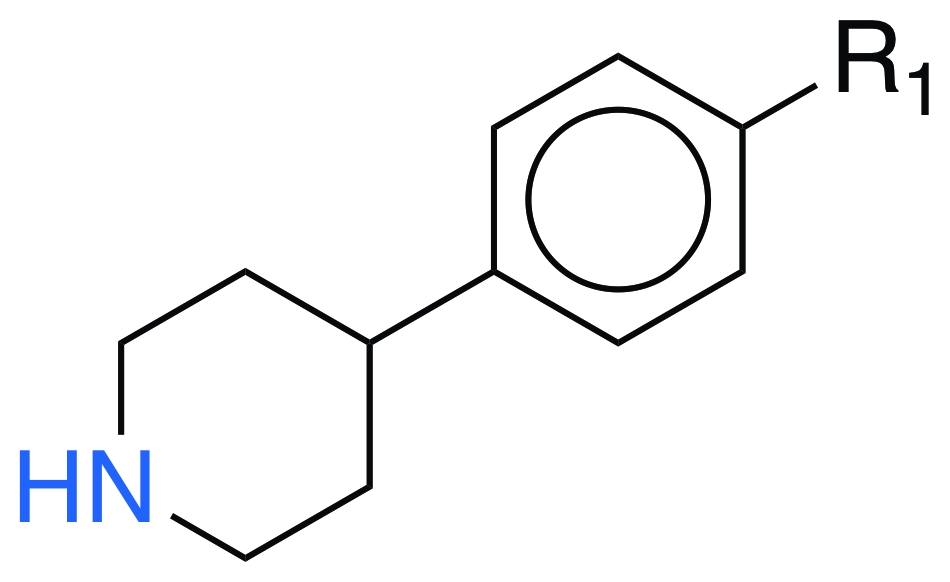 | 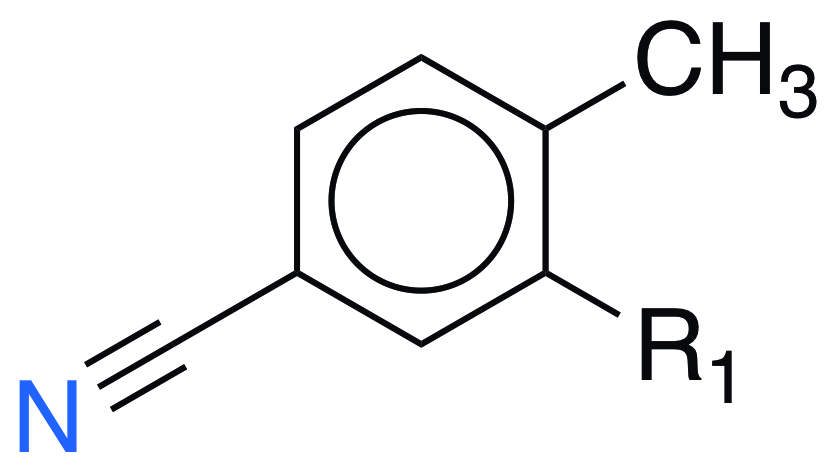 | 3.2 | 63.9 |
| 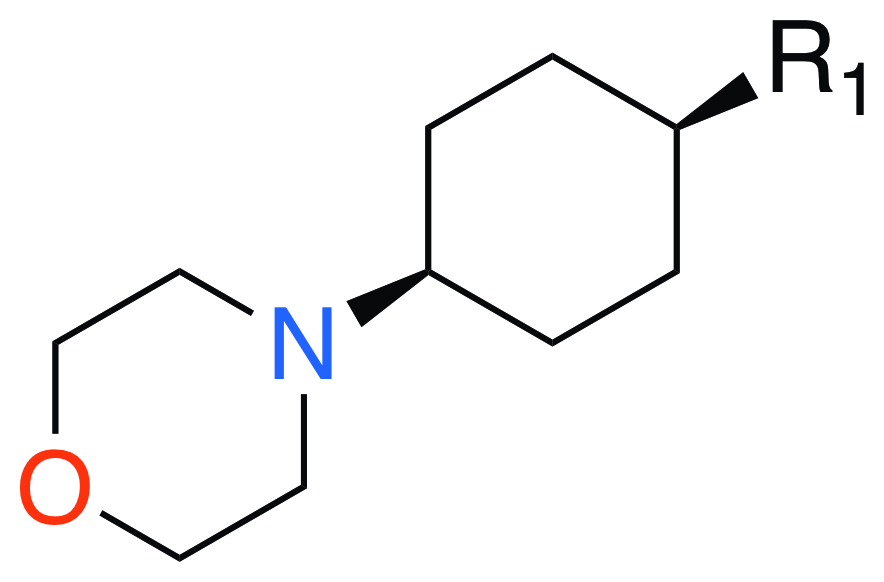 | 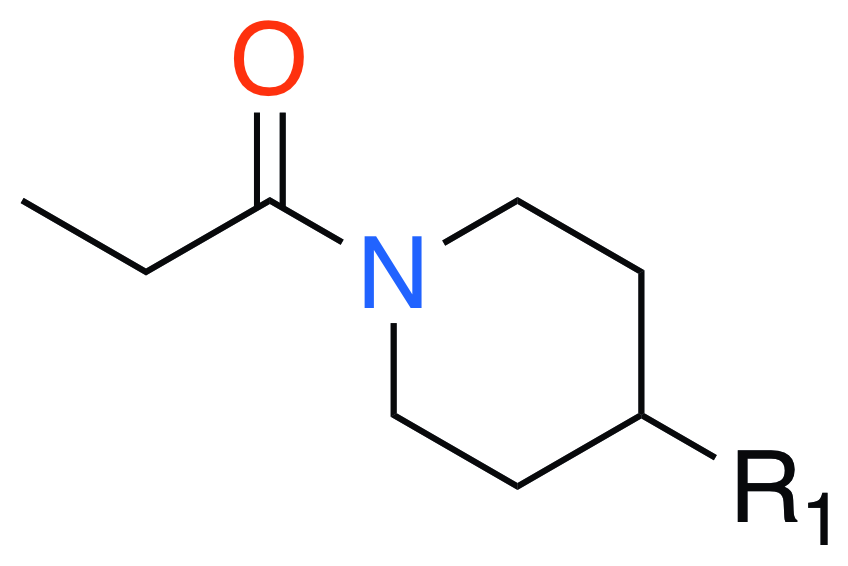 | 14.1 | >120.0 |
| 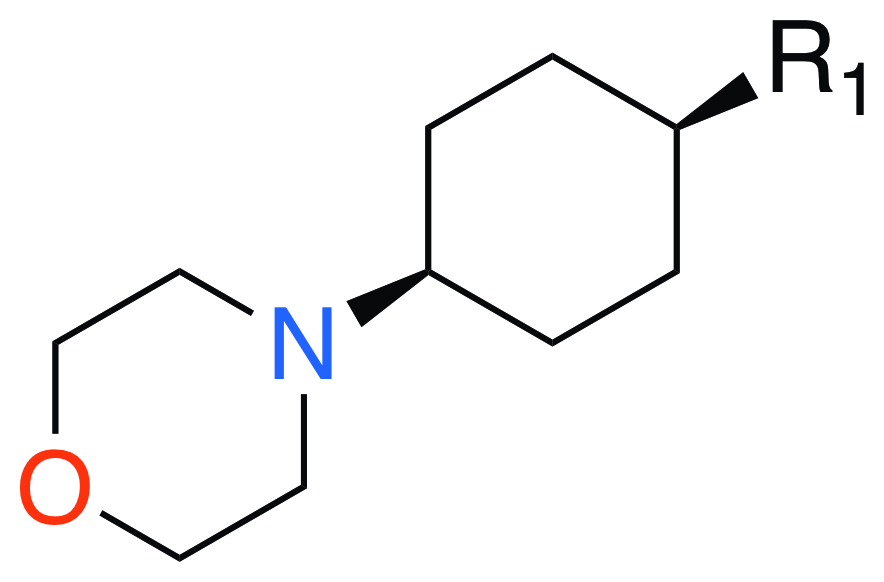 | 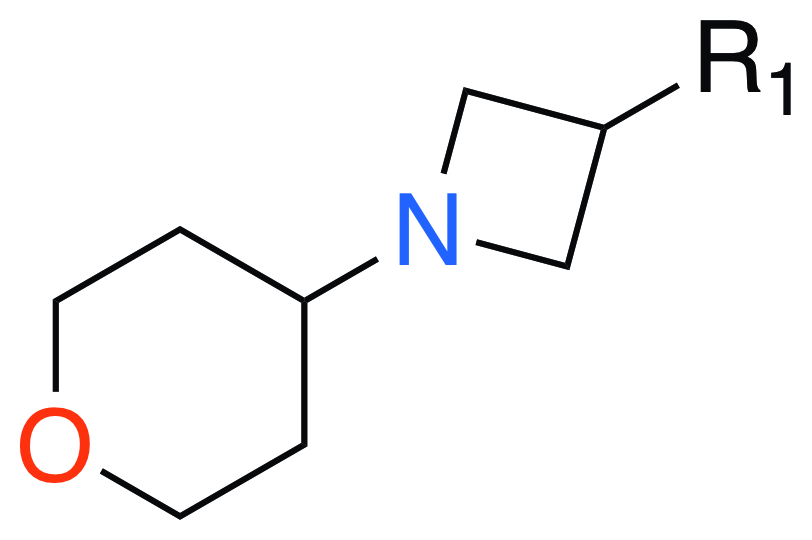 | 14.1 | >120.0 |
| 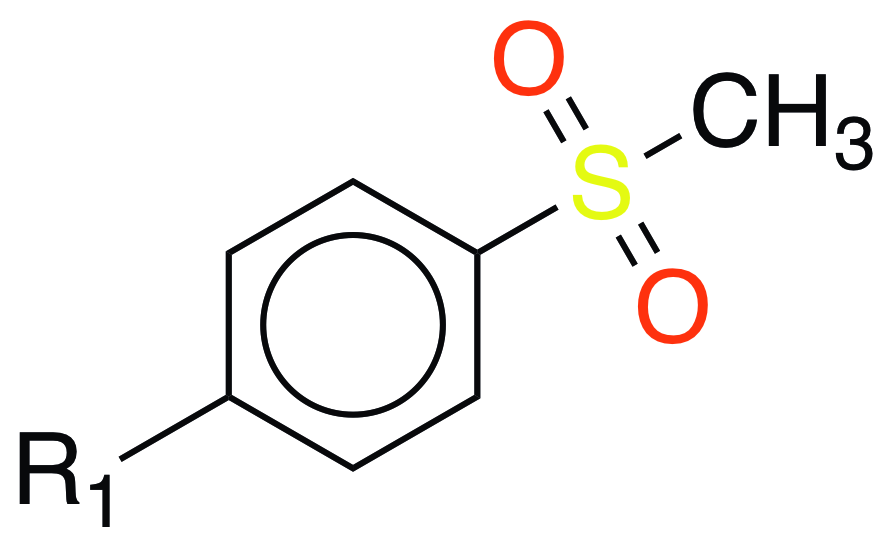 | 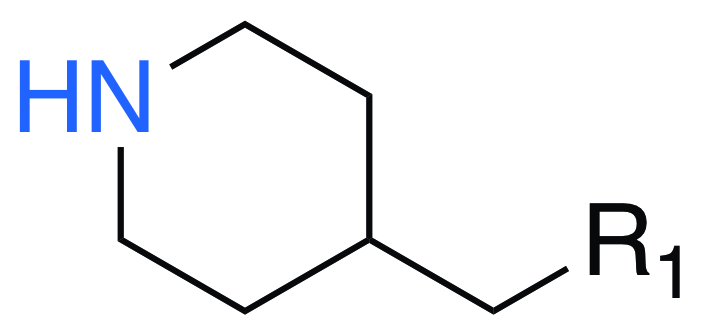 | 14.7 | >120.0 |
| 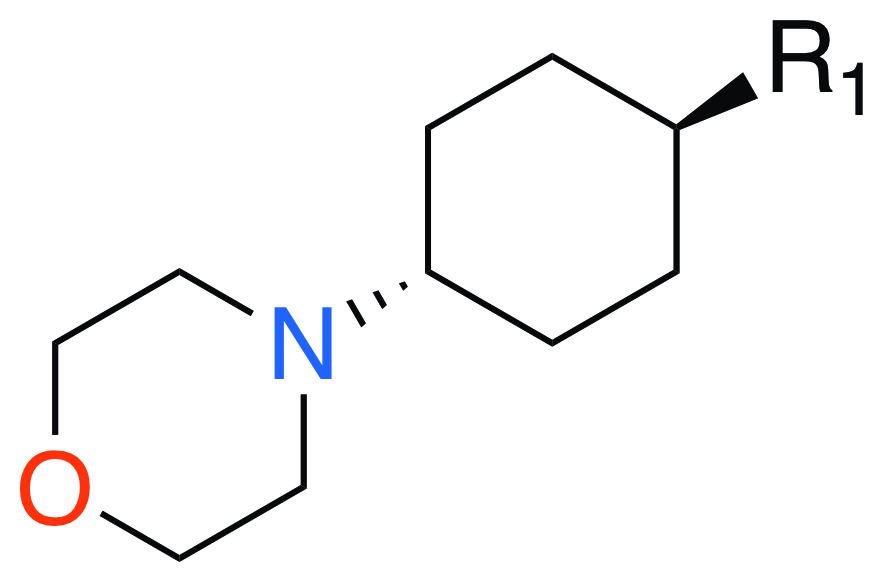 | 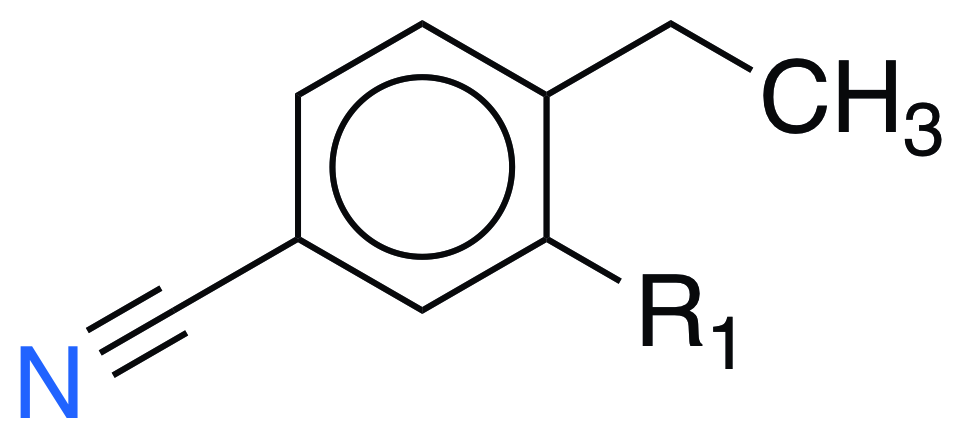 | 17.4 | >120.0 |
| 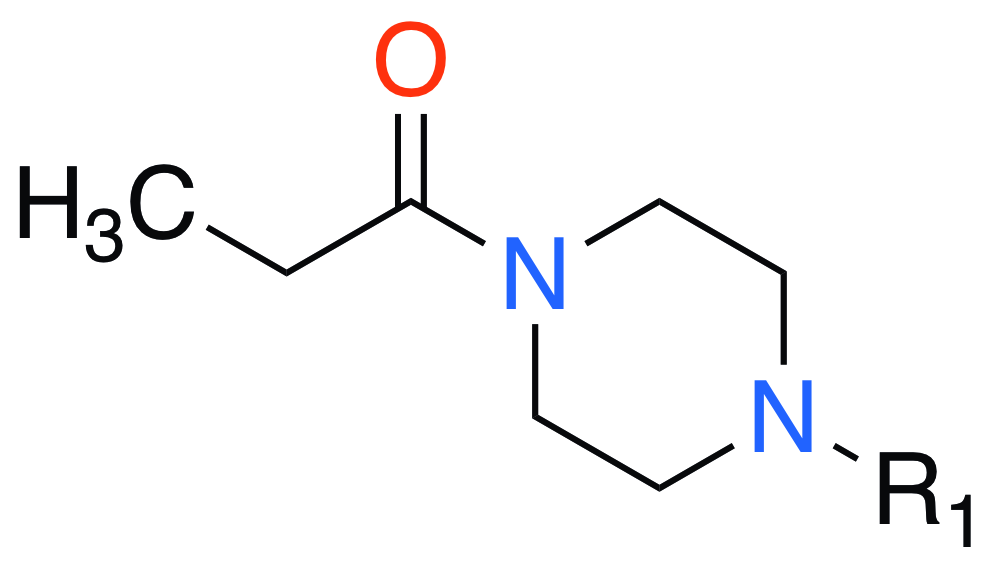 | 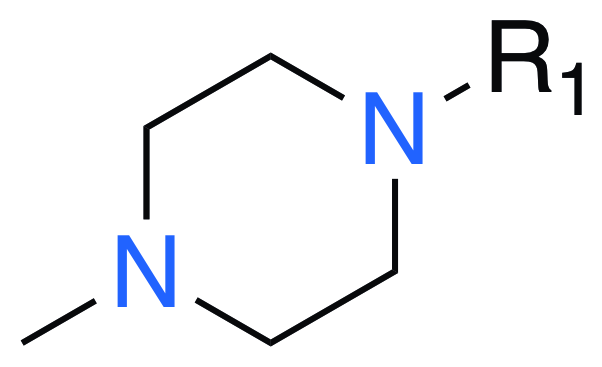 | 13.8 | 89.8 |
| 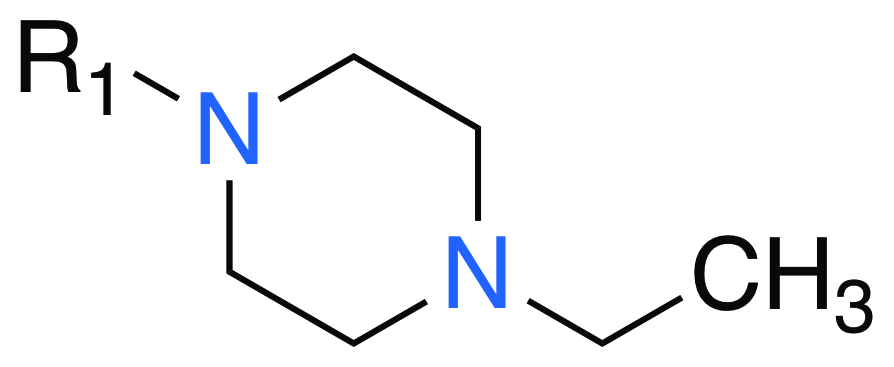 | 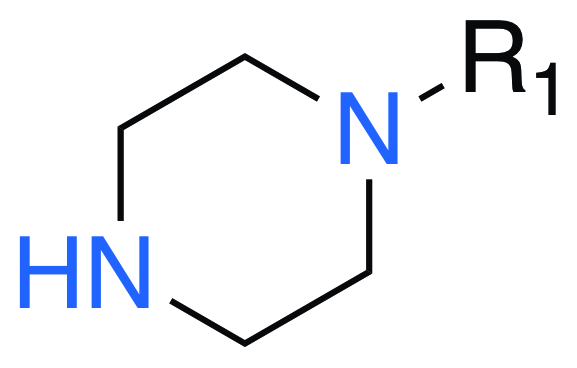 | 20.2 | >120.0 |

**Table S3**. Top 10 chemical transformations from mouse training dataset that successfully transformed an unstable compound in the test dataset to a stable compound.

| **Left Fragment** | **Right Fragment** | **t_1/2_ [min] (Left)** | **t_1/2_ [min] (Right)** |
| --- | --- | --- | --- |
| 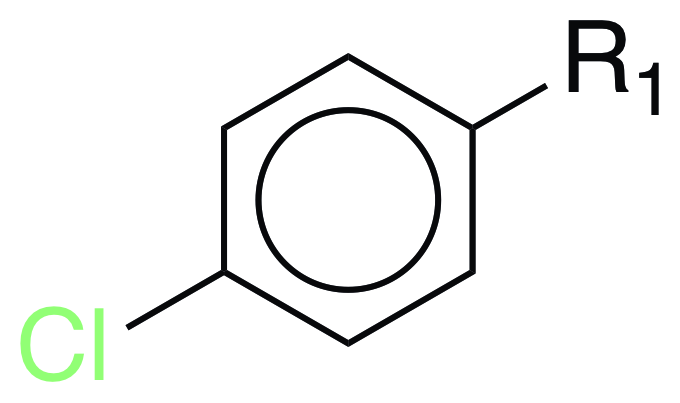 | 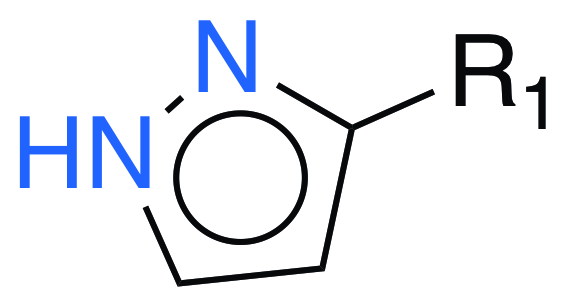 | 20.7 | >120.0 |
| 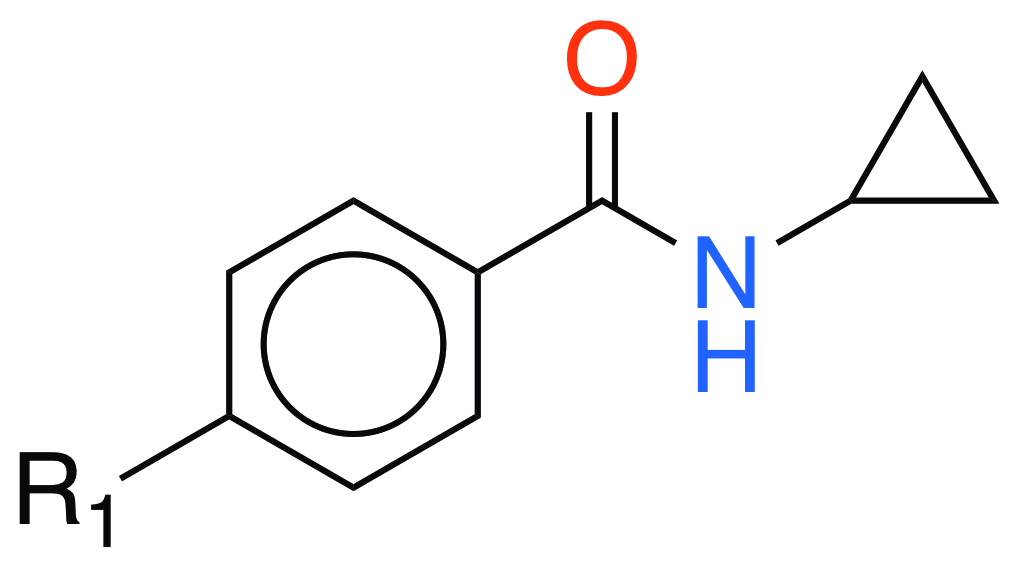 | 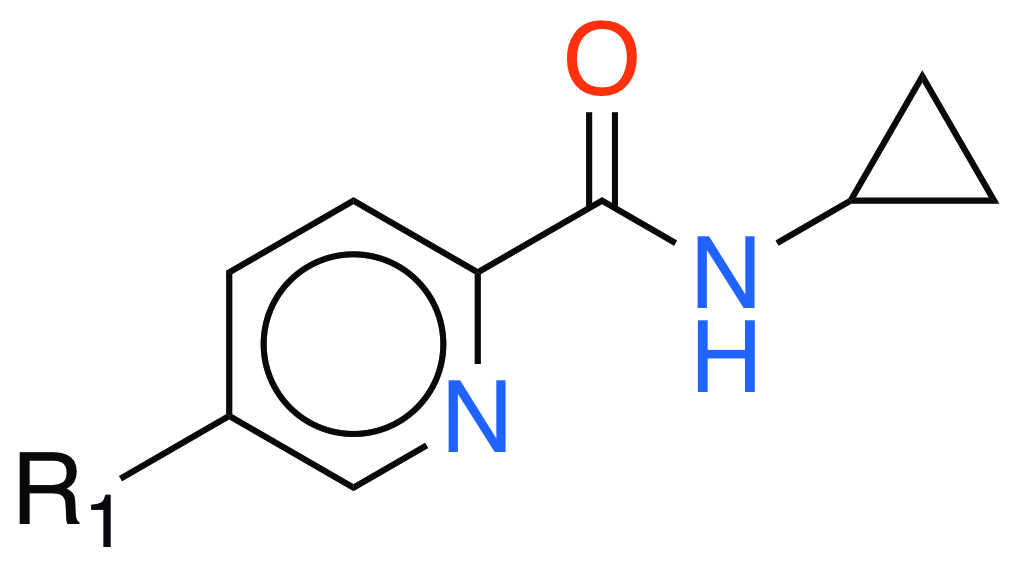 | 13.0 | 73.7 |
| 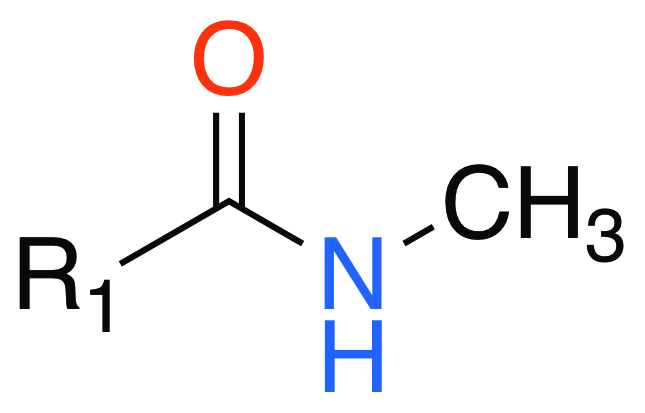 | 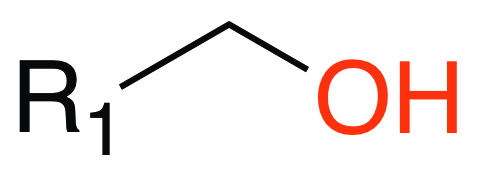 | 26.9 | >120.0 |
| 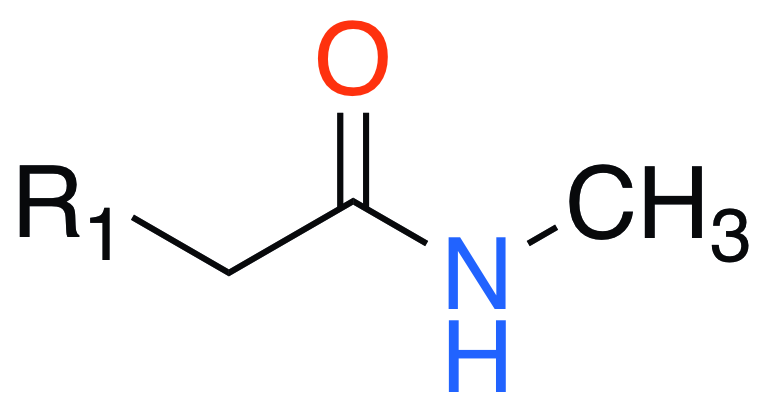 | 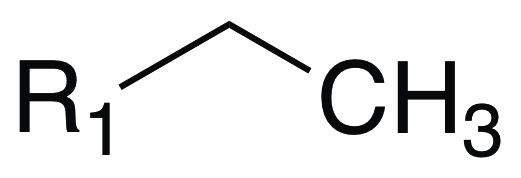 | 26.9 | >120.0 |
| 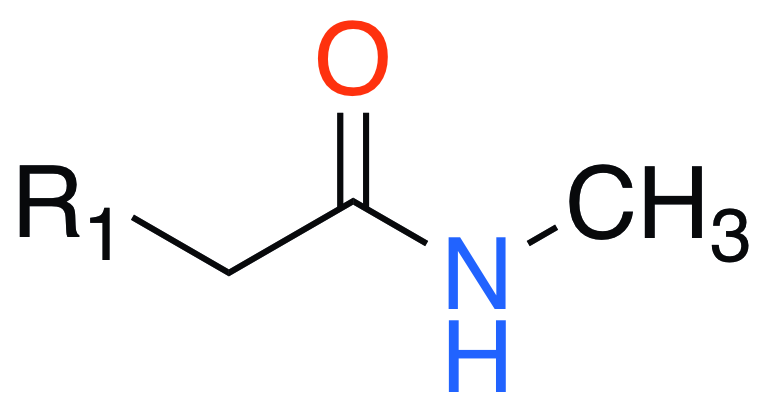 | 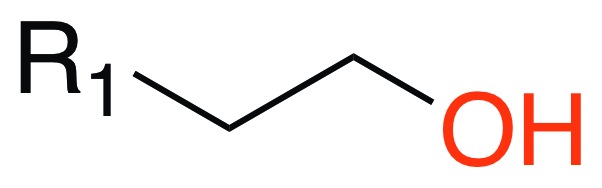 | 26.9 | >120.0 |
| 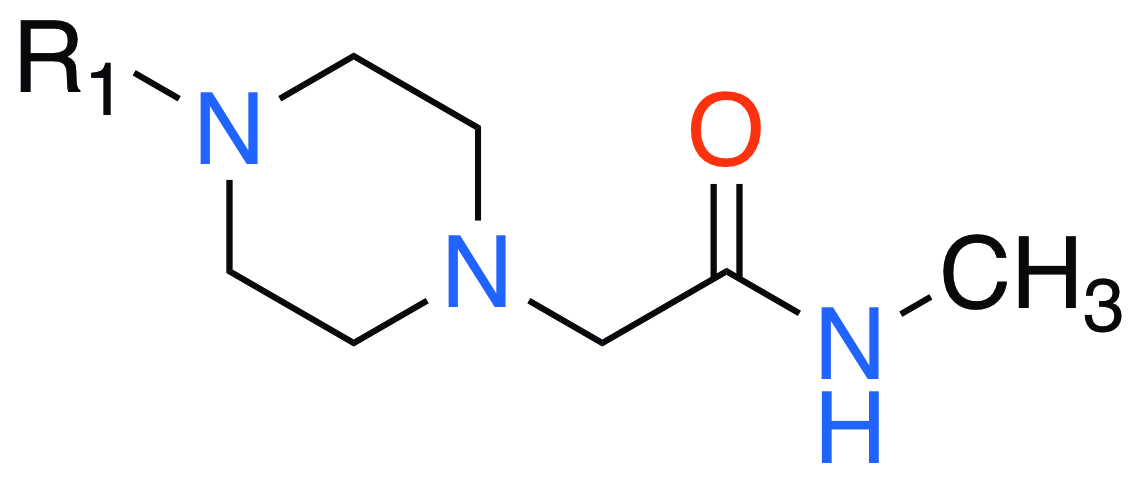 | 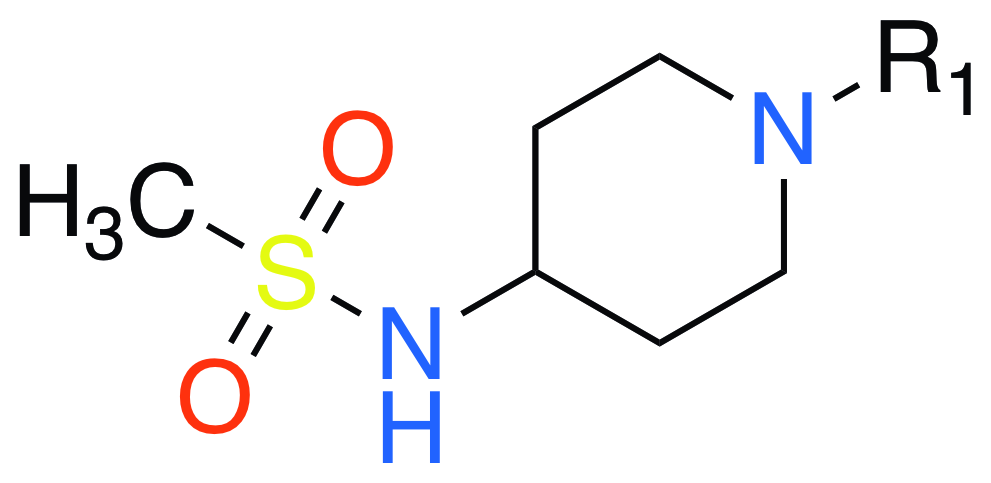 | 26.9 | >120.0 |
| 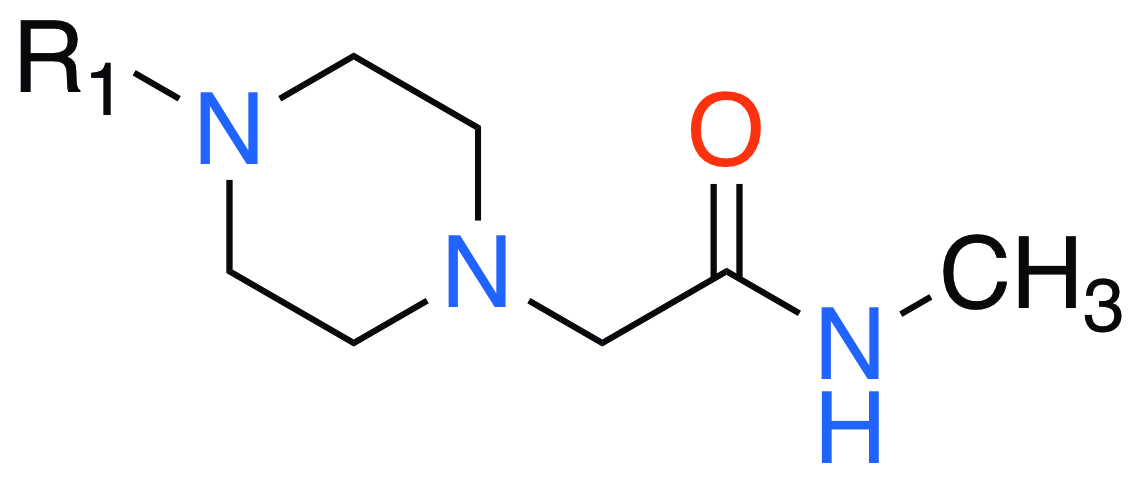 | 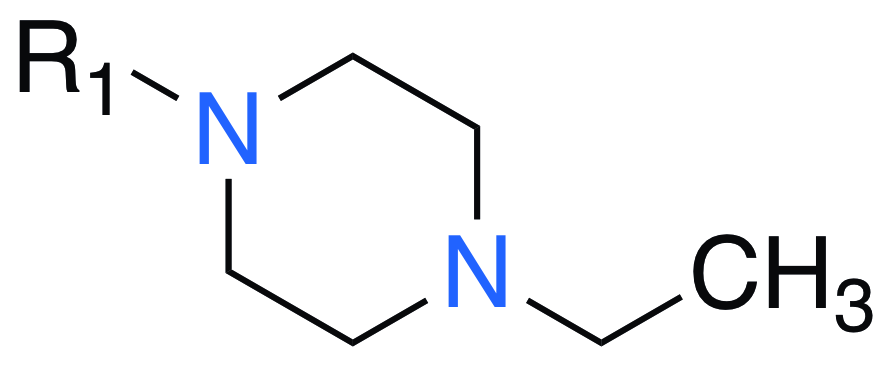 | 26.9 | >120.0 |
| 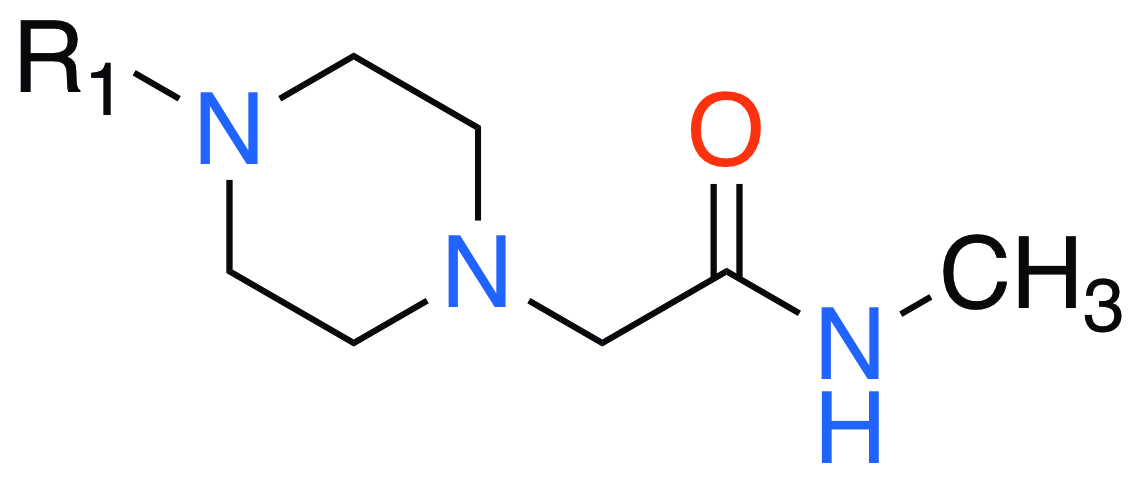 | 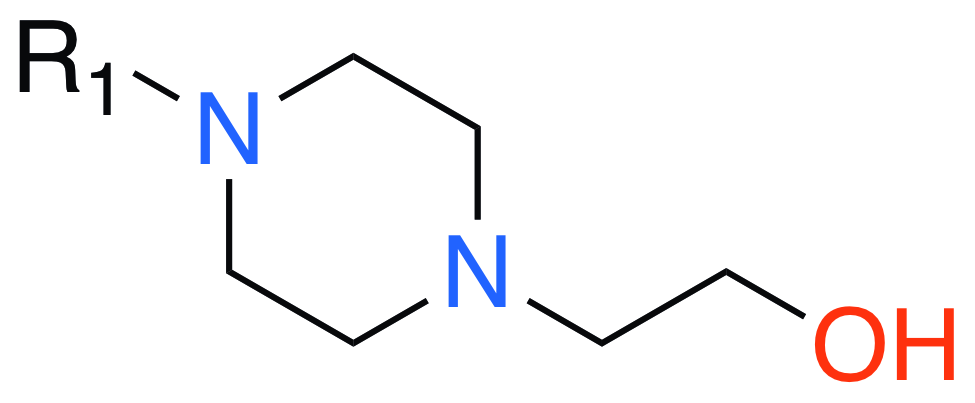 | 26.9 | >120.0 |
| 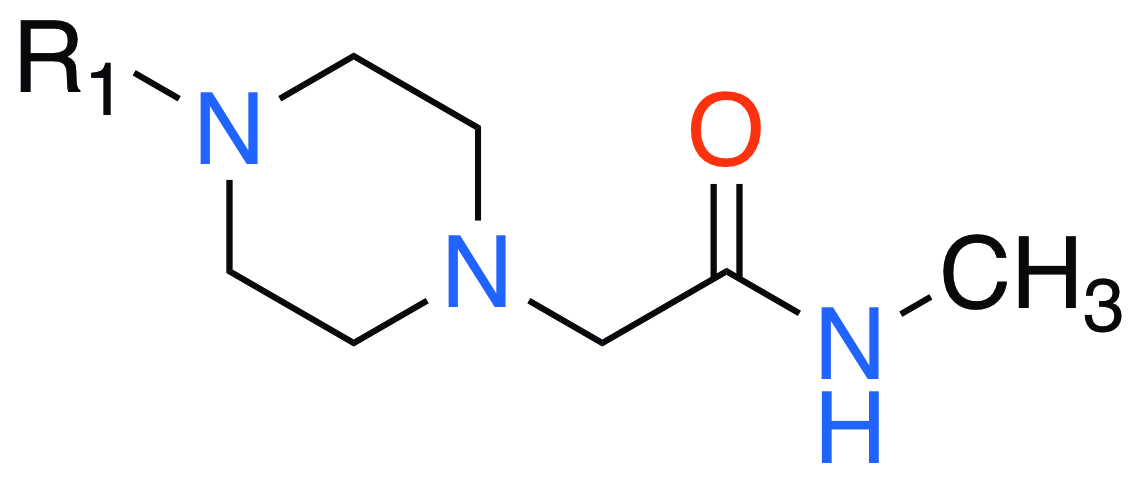 | 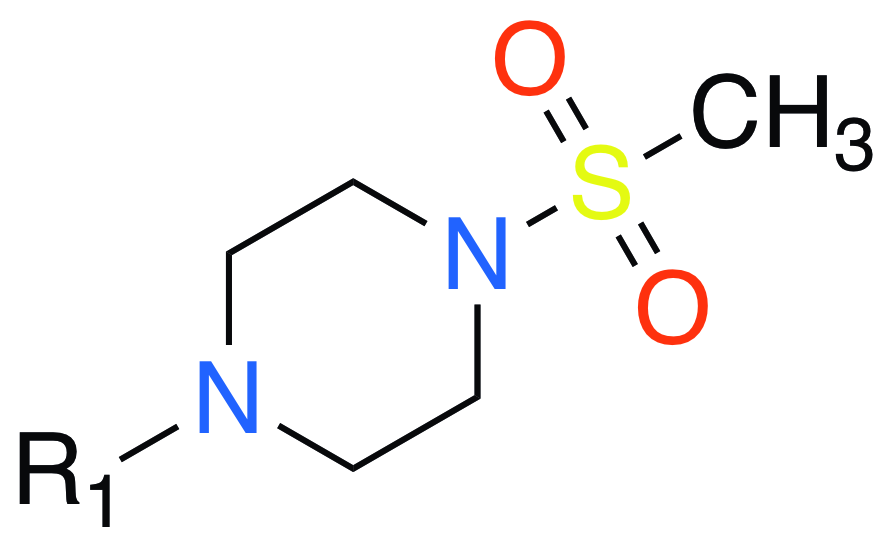 | 26.9 | >120.0 |
| 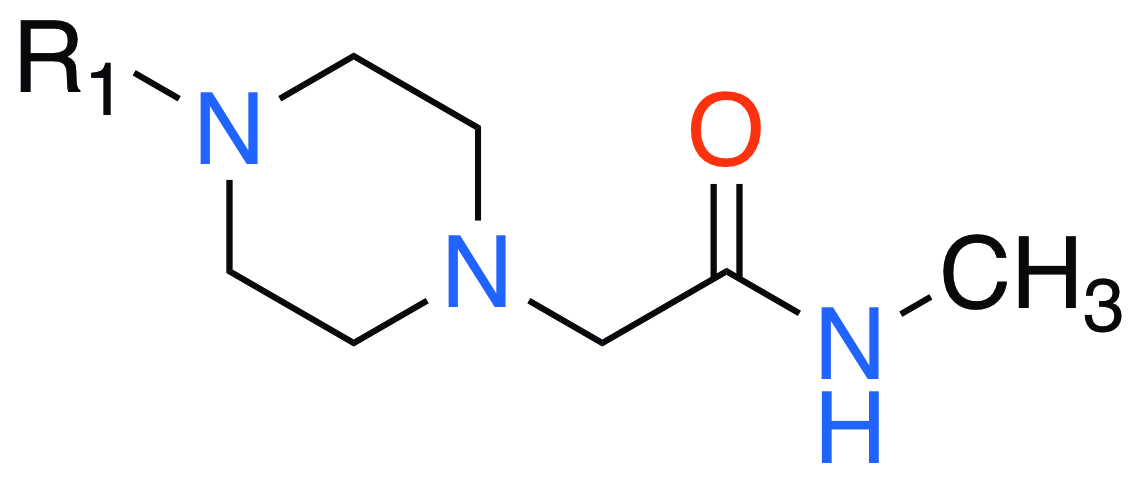 | 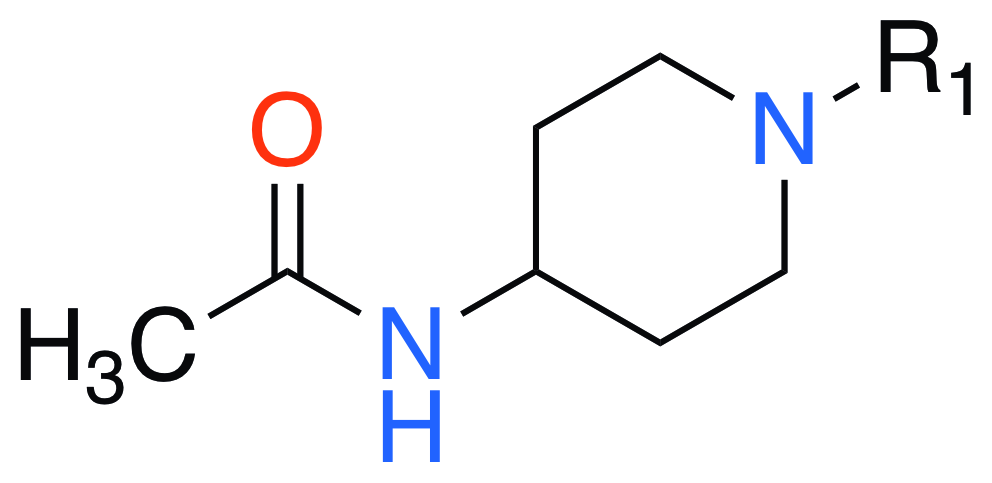 | 26.9 | 93.3 |

**Table S4**. Modeling parameters for different machine learning algorithms employed in this study.

| **Algorithm** | **Parameters & Other Details** |
| --- | --- |
| **Random Forests** | No. of trees: *100*  Split criterion: *Information Gain*  Static Random Seed: *Yes* |
| **SVM** | Kernel: *Polynomial*  Bias: *1.0*  Power: *1.0*  Gamma: *1.0* |
| **BayesNet** | Estimator: *SimpleEstimator*  Search Algorithm: *K2*  ADTree: *Not Used* |
| **ChemProp - Graph Convolutional Networks** | Activation function: ReLu  LR_decay_rate: 0.9  Epochs: 30  Batch size: 50 |

**Table S5**. Performance metrics employed in validation of the QSAR models.

| **Performance Metric** | **Formula** |
| --- | --- |
| **Sensitivity**: Accuracy of predicting “positive” when the true outcome is positive. | $Sensitivity=TP/(FN+TP)$ |
| **Specificity**: Accuracy of predicting “negative” (inactive) when the true outcome is negative. | $Specificity=TN/(TN+FP)$ |
| **Balanced Accuracy (BACC)**:  An average of Sensitivity and Specificity. | $BACC=(Sensitivity+Specificity)/2$ |
| **Area under the ROC curve (AUC-ROC)**: ROC curve plots the true positive rate against the false positive rate. AUC-ROC provides an estimate of the quality of classification. | Not Applicable |

TP, FP, TN, FN are True Positive, False Positive, True Negative and False Negative predictions, respectively.

**Table S6**. Complete cross-validation results for the cytosol stability prediction models.

| **Descriptor** | **Method*^a^*** | **AUC-ROC** | **BACC** | **Sensitivity** | **Specificity** |
| --- | --- | --- | --- | --- | --- |
| **MOE** | BayesNet | 0.81 | 0.72 | 0.62 | 0.81 |
|  | BayesNet - DU | **0.81** | **0.76** | **0.72** | **0.79** |
|  | BayesNet - MU | 0.81 | 0.72 | 0.72 | 0.72 |
|  | RF | 0.87 | 0.75 | 0.57 | 0.94 |
|  | RF - DU | 0.87 | 0.76 | 0.62 | 0.91 |
|  | RF - MU | **0.86** | **0.78** | **0.79** | **0.76** |
|  | SVM | 0.85 | 0.71 | 0.47 | 0.95 |
|  | SVM - DU | **0.85** | **0.77** | **0.79** | **0.76** |
|  | SVM - MU | 0.84 | 0.77 | 0.79 | 0.76 |
| **RDKit** | BayesNet | **0.79** | **0.73** | **0.61** | **0.85** |
|  | BayesNet - DU | 0.81 | 0.72 | 0.64 | 0.80 |
|  | BayesNet - MU | 0.79 | 0.71 | 0.68 | 0.75 |
|  | RF | 0.87 | 0.73 | 0.54 | 0.92 |
|  | RF - DU | 0.87 | 0.76 | 0.62 | 0.91 |
|  | RF - MU | **0.86** | **0.78** | **0.78** | **0.78** |
|  | SVM | 0.83 | 0.67 | 0.38 | 0.95 |
|  | SVM - DU | **0.83** | **0.77** | **0.83** | **0.72** |
|  | SVM - MU | 0.83 | 0.76 | 0.82 | 0.71 |
| **QNA** | BayesNet | 0.75 | 0.68 | 0.59 | 0.77 |
|  | BayesNet - DU | 0.73 | 0.68 | 0.61 | 0.74 |
|  | BayesNet - MU | **0.75** | **0.69** | **0.62** | **0.75** |
|  | RF | 0.84 | 0.70 | 0.44 | 0.95 |
|  | RF - DU | 0.85 | 0.72 | 0.52 | 0.93 |
|  | RF - MU | **0.83** | **0.75** | **0.72** | **0.78** |
|  | SVM | 0.84 | 0.73 | 0.52 | 0.93 |
|  | SVM - DU | **0.85** | **0.80** | **0.80** | **0.80** |
|  | SVM - MU | 0.84 | 0.77 | 0.78 | 0.77 |

*^a^* *DU and MU stand for diversity under-sampling and multiple under-sampling, respectively*

**Table S7**. Complete external validation results for the cytosol stability prediction models.

| **Descriptor** | **Method** | **AUC-ROC** | **BACC** | **Sensitivity** | **Specificity** |
| --- | --- | --- | --- | --- | --- |
| **MOE** | BayesNet | **0.62** | **0.60** | **0.75** | **0.45** |
|  | BayesNet - DU | 0.57 | 0.55 | 0.19 | 0.91 |
|  | BayesNet - MU | 0.57 | 0.51 | 0.71 | 0.31 |
|  | RF | **0.73** | **0.64** | **0.63** | **0.66** |
|  | RF - DU | 0.67 | 0.67 | 0.56 | 0.78 |
|  | RF - MU | 0.61 | 0.56 | 0.60 | 0.51 |
|  | SVM | 0.51 | 0.53 | 0.06 | 0.99 |
|  | SVM - DU | 0.53 | 0.55 | 0.13 | 0.98 |
|  | SVM - MU | **0.51** | **0.56** | **0.35** | **0.86** |
| **RDKit** | BayesNet | **0.56** | **0.57** | **0.50** | **0.64** |
|  | BayesNet - DU | 0.63 | 0.58 | 0.19 | 0.98 |
|  | BayesNet - MU | 0.53 | 0.50 | 0.71 | 0.29 |
|  | RF | 0.66 | 0.60 | 0.44 | 0.77 |
|  | RF - DU | 0.69 | 0.53 | 0.13 | 0.94 |
|  | RF - MU | **0.68** | **0.61** | **0.71** | **0.52** |
|  | SVM | 0.46 | 0.50 | 0.00 | 1.00 |
|  | SVM - DU | 0.59 | 0.48 | 0.00 | 0.96 |
|  | SVM - MU | **0.62** | **0.54** | **0.15** | **0.94** |
| **QNA** | BayesNet | **0.63** | **0.62** | **0.63** | **0.60** |
|  | BayesNet - DU | 0.68 | 0.55 | 0.44 | 0.66 |
|  | BayesNet - MU | 0.61 | 0.57 | 0.71 | 0.43 |
|  | RF | 0.62 | 0.55 | 0.19 | 0.91 |
|  | RF - DU | **0.50** | **0.58** | **0.25** | **0.89** |
|  | RF - MU | 0.53 | 0.52 | 0.58 | 0.45 |
|  | SVM | 0.59 | 0.56 | 0.25 | 0.88 |
|  | SVM - DU | 0.60 | 0.56 | 0.31 | 0.81 |
|  | SVM - MU | **0.60** | **0.57** | **0.63** | **0.52** |
| **Graphs** | GCN | 0.48 | 0.53 | 0.38 | 0.68 |
|  | GCN - DU | 0.36 | 0.44 | 0.00 | 0.88 |
|  | GCN – MU | 0.44 | 0.50 | 0.10 | 0.89 |

**Figure S1**. PCA plots for compounds from training and validation datasets based on different molecular descriptors: MOE, RDKit and QNA. Color coding: purple – validation set compounds; green – stable compounds from training set; red – unstable compounds from training set.
